# Hypertrophic chondrocytes at the junction of musculoskeletal structures

**DOI:** 10.1016/j.bonr.2023.101698

**Published:** 2023-07-03

**Authors:** Ning Chen, Robin W.H. Wu, Yan Lam, Wilson C.W. Chan, Danny Chan

**Affiliations:** a School of Biomedical Sciences, The University of Hong Kong, Hong Kong, China; b Department of Orthopaedics Surgery and Traumatology, The University of Hong Kong-Shenzhen Hospital (HKU-SZH), Shenzhen 518053, China

**Keywords:** Chondrocyte hypertrophy, Transdifferentiation, Endochondral ossification, Cellular plasticity, ECM remodeling

## Abstract

Hypertrophic chondrocytes are found at unique locations at the junction of skeletal tissues, cartilage growth plate, articular cartilage, enthesis and intervertebral discs. Their role in the skeleton is best understood in the process of endochondral ossification in development and bone fracture healing. Chondrocyte hypertrophy occurs in degenerative conditions such as osteoarthritis. Thus, the role of hypertrophic chondrocytes in skeletal biology and pathology is context dependent. This review will focus on hypertrophic chondrocytes in endochondral ossification, in which they exist in a transient state, but acting as a central regulator of differentiation, mineralization, vascularization and conversion to bone. The amazing journey of a chondrocyte from being entrapped in the extracellular matrix environment to becoming proliferative then hypertrophic will be discussed. Recent studies on the dynamic changes and plasticity of hypertrophic chondrocytes have provided new insights into how we view these cells, not as terminally differentiated but as cells that can dedifferentiate to more progenitor-like cells in a transition to osteoblasts and adipocytes, as well as a source of skeletal stem and progenitor cells residing in the bone marrow. This will provide a foundation for studies of hypertrophic chondrocytes at other skeletal sites in development, tissue maintenance, pathology and therapy.

## Highlights

• Hypertrophic chondrocytes exist in various junctions between soft and ossified tissues.
• Diverse molecular and metabolic controls regulate the lineage and fate of hypertrophic chondrocytes.
• Hypertrophic chondrocytes play key roles in orchestrating and fine-tuning the process of endochondral ossification.
• Chondrocytes are highly plastic and can transition into osteoblasts and adipocytes via dedifferentiation and redifferentiation.

## Graphical abstract

**Figure:**
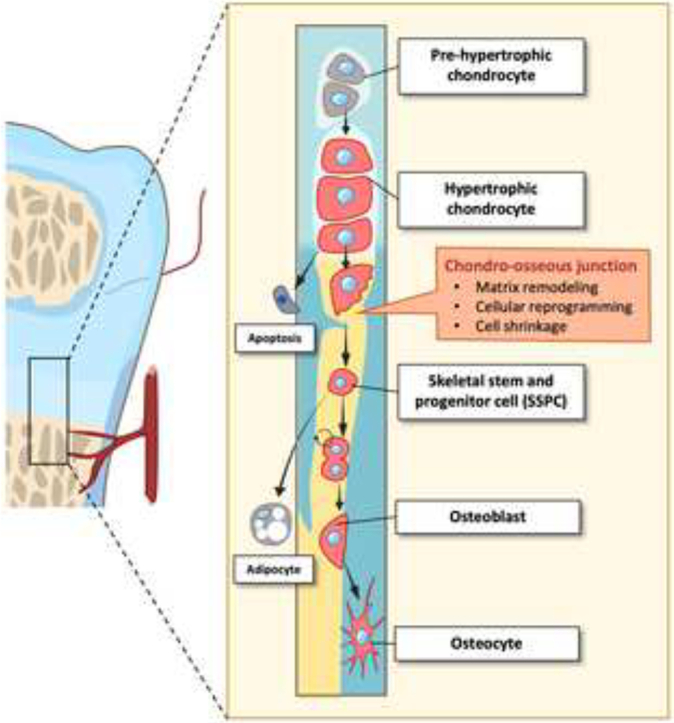

## 1 Introduction

Cells at the intersection between two different types of tissues are special. These cells are often dualistic, having characteristics of adjacent cell types, and play vital roles in the structural connection and functional balance of the two tissues (Garcia et al., 2018). A good example is the myotendinous junction at the tendon and muscle interface (Schejter and Baylies, 2010). This junction contains sarcolemma interdigitation, formed by connection of muscle membrane and the collagen fibrils from tendon. This unique structure between the two tissue types significantly increases the force transfer area at the junction between muscles and tendons, resulting in better force distribution and reduced localized stress (Tidball and Lin, 1989). Similarly, the enthesis, a fibrocartilaginous tissue, provides stable anchoring function to connect tendon and bone (Benjamin and McGonagle, 2009). The enthesis is lined with distinct cellular zones, with a gradational change from tenocytes, uncalcified fibrochondrocytes, calcified fibrochondrocytes, to osteocytes, in the direction from the tendon to the bone. This orderly arrangement of different cellular regions allows for a connection between tendon and bone at the cellular level (Benjamin et al., 2002). It is clear that cells at tissue junctions have key roles in mediating the development and maintenance of tissue function, sparking an interest as to the intricacy and complexity of cell regulation in this context.

Hypertrophic chondrocytes are one such type of junctional cells that are found between non-mineralized cartilage and the hard ossified bone in many skeletal structures, such as the cartilage growth plates of long bones (Fernández-Iglesias et al., 2021), the articular cartilage of synovial joints (Gauci et al., 2019), and the tendon/bone insertion sites (Killian, 2022). Hypertrophic chondrocytes are characterized morphologically as enlarged chondrocytes. Molecularly, they specifically express *Col10a1*, encoding type X Collagen, an extracellular matrix (ECM) protein (Kong et al., 1993). While the function of type X Collagen is still not well understood, this gene is a key marker for hypertrophic chondrocytes, and has been used in the generation of genetic tools in the mouse to study the role and fate of hypertrophic chondrocytes in the mammalian system, in particular, in the cartilage growth plate (Warman et al., 1993; Gu et al., 2014). Hypertrophic chondrocytes have also been used as a marker for degenerative conditions such as osteoarthritis (Park et al., 2021). This review will focus on this unique cell type, and our current understanding of its characteristics and roles in the context of its presence and interplay with adjacent cells at specific junction sites.

## 2 Hypertrophic chondrocytes in the mammalian skeletal system

**Fig. 1:**
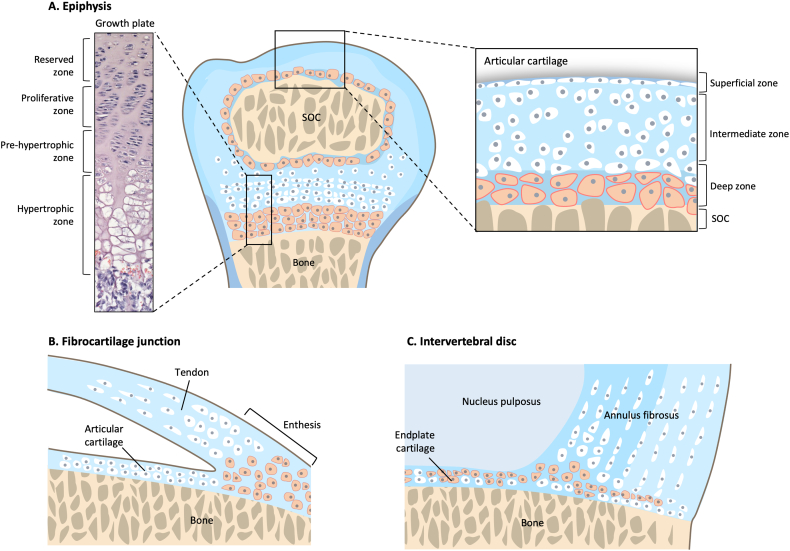
Schematic diagram for the location of hypertrophic chondrocytes in the skeletal system Hypertrophic chondrocytes are located at multiple sites in the skeletal system, particularly at tissue junctions. They exist as transient cells in the cartilage growth plate at the ends of long bone, at the periphery of the secondary ossification centre (SOC) of the epiphysis end which separates the articular cartilage from the growth plate (A), the fibrocartilage junction at the tendon insertion site (enthesis) to cartilage/bone (B), in the intervertebral disc within the cartilage endplate flanking the inner annulus fibrosus (C). Hypertrophic chondrocytes are coloured in orange.

The characteristics of hypertrophic chondrocytes are best described in the context of the growth plate cartilage by light and electron microscopy, which reveal distinct stages of these “terminal hypertrophic chondrocytes” (Farnum and Wilsman, 1987) (Fig. 1). The majority of these cells exist as fully hydrated cells with an intact plasma membrane in direct contact with the pericellular matrix. Some are found as condensed cells that retain attachment to the last transverse septum and, interestingly, about 1 % of these cells consistently have a direct asymmetrical contact of the plasma membrane with the last transverse septum (Farnum and Wilsman, 1987). Thus, in the context of the growth plate cartilage, which is a transitional cartilage with dynamic changes, heterogeneity exist in this population of hypertrophic chondrocytes (Long et al., 2022).

While articular cartilage is considered as a stable cartilage, it shows a degree of spatial and temporal transiency, with defined superficial, intermediate and deep zones, and their associated cellular changes (Fig. 1). The superficial zone contains flattened chondrocytes aligned parallel to the surface of the cartilage, where they express ECM components that facilitate lubrication, such as lubricin (PRG4) (Kozhemyakina et al., 2015b). The intermediate zone contains chondrocytes with differing zonal expression of type II collagen and aggrecan. The deep zone contains hypertrophic chondrocytes that express type X collagen (Chau et al., 2022). Type X collagen/*COL10A1* as a specific protein/gene maker for hypertrophic chondrocytes has facilitated the identification of hypertrophic chondrocytes at other skeletal junction sites, including the Achilles tendon to the calcaneus/bone (Fukuta et al., 1998; Taylor et al., 2020), the femoral insertion of the medial collateral ligament (Niyibizi et al., 1996), and the intervertebral disc (Boneski et al., 2022) (Fig. 1). Furthermore, abnormal chondrocyte hypertrophy is implicated as part of the pathology seen in many skeletal dysplasias and osteoarthritis (OA) (Park et al., 2021). Thus, hypertrophic chondrocytes are involved in skeletal development, tissue homeostasis and disease states.

## 3 Type X collagen and hypertrophic chondrocytes

Studies on the expression and localization of type X collagen suggest the characteristics of hypertrophic chondrocytes at various transitional zones are not the same. *Col10a1* expression follows the fate of hypertrophic chondrocyte. In endochondral ossification, such as during the developmental changes in the growth plate, fracture repair, and heterotopic ossification (Grant et al., 1987; Rooney et al., 1992), expression of *Col10a1* is transient. In this context, type X collagen is degraded with ossification. By contrast, type X collagen persists at the enthesis (Niyibizi et al., 1996) and articular cartilage through maturity (Fujioka et al., 1997), with the presence of hypertrophic chondrocytes. Immunostaining for type X collagen indicates a primarily pericellular localization in most sites, for example for tendon attachment in rat and turkey (Fujioka et al., 1997; Taylor et al., 2020), and in the territorial matrix at sites of secondary ossification (Eerola et al., 1998). Whether this reflects a real difference in hypertrophic chondrocyte characteristics or technical variations remains to be resolved. However, it is clear that the presence of type X collagen in the ECM is an indicator of the progressive changes in growth plate during endochondral ossification, demonstrating a role for type X collagen and hypertrophic chondrocytes in skeletal health and disease. In OA progression, chondrocyte hypertrophy is thought to be associated with high apoptotic rates and presence of type X collagen as an indicator of cartilage destruction (van der Kraan et al., 2009; van der Kraan and van den Berg, 2012).

To date, the function of type X collagen remains elusive. Two studies on inactivation of the *Col10a1* gene in mice showed no (Rosati et al., 1994) or subtle (Kwan et al., 1997) phenotypic changes in skeletal development, such as mildly reduced thickness of the cartilage growth plate and articular cartilage, some alteration in bone content and incidence of mild *coxa vara* in older mice. Whereas, over-expression of a truncated chicken type X collagen in a transgenic mouse also resulted in a dwarfism phenotype, and immunological defects were noted with leukocyte deficiency in bone marrow, reduction in size of thymus and spleen, and lymphopenia (Jacenko et al., 1993). Thus, *Col10a1* is dispensable and not required for chondrocyte hypertrophy, but there is some evidence for its role as a negative regulator of mineralization and the distribution of matrix vesicles into the mineralizing zone from the chondro-osseous junction (Kwan et al., 1997). Interestingly, mutations in *COL10A1* result in metaphyseal chondrodysplasia type Schmid (MCDS, OMIM 156500), a form of dwarfism in which bone growth is disrupted (Ikegawa et al., 1998). Later studies confirmed that the effects of these mutations are not due to a loss-of-function outcome, but rather, a dominant-negative effect on the assembly and secretion of type X collagen, impairing maintenance and differentiation of hypertrophic chondrocytes (Chan et al., 1995; Chan et al., 1996). Thus, the presence, fate and clearance of hypertrophic chondrocytes have contrasting roles in normal endochondral ossification and diseases, and targeting these cells could have therapeutic potential (Park et al., 2021). Further, given the active turnover of the mineralized cartilage in linear bone growth, a degradative product, the noncollagenous 1 (NC1) domain, of type X collagen can be detected in the circulation in early human growth, that can be used as a marker for child health and development; in particular, period of active bone growth (Coghlan et al., 2017). As bone growth is not linear, such information could allow more precise treatments and monitoring of bone growth defects.

## 4 Chondrocyte hypertrophy in the initiation of endochondral ossification

The axial and appendicular bones are formed through endochondral ossification. This is a complex process, involving the generation of cartilage templates that are subsequently converted to bone. Many cell types are involved, and hypertrophic chondrocytes located in the region of the calcified cartilage play a key role in the conversion to bone, in distinct and closely coupled steps. It begins with chondrocyte hypertrophy and formation of the primary ossification center in the cartilage templates; cells in the surrounding perichondrium differentiate into osteoblasts, forming a bone collar-like structure (Kronenberg, 2003). This is followed by invasion of the calcified cartilage by blood vessels, bringing in pericytes, osteoclasts and progenitor cells, converting the calcified cartilage into vascularized bony trabeculae.

Primary ossification progresses towards both ends of the cartilage template, and a growth plate is established, where new cartilage is generated for the conversion to bone, thus, allowing the linear growth of long bones. The cartilage growth plate is an organized structure containing four distinct zones based on status of the chondrocytes: reserve (also termed “resting” in other literature), proliferative, pre-hypertrophic, and hypertrophic zones. The molecular interplay between the cells in these zones are tightly regulated for proper bone growth, the disruption of which will lead to various forms of skeletal dysplasia (Kozhemyakina et al., 2015a). During development, secondary ossification centers initiate at both cartilage ends within the reserve zones. This process is similar to the initiation of the primary ossification center, spatially separates the growth plate and the articular cartilage with trabecular bone. The hypertrophic chondrocytes from this secondary ossification center contribute to the organizational structure of the articular cartilage, as part of the deep calcified zone (Fig. 1), whereas chondrocytes in the superficial and intermediate zones are derived from progenitor cells within the interzone during synovial joint development (Feng et al., 2019). Lineage studies show interzone cells do contribute in part to the hypertrophic chondrocytes located in the deep zone (Feng et al., 2019). Thus, hypertrophic chondrocytes in the deep zone may have multiple origins. Interestingly, hypertrophic chondrocytes present in the carpal and tarsal bones of the fore and hind limbs are derived through a process similar to the formation of secondary ossification centers (Lazarus et al., 2017).

## 5 Role of pre-hypertrophic chondrocytes in regulating bone growth

The structural organization of the cartilage growth plate is unique, with distinct zones that are intertwined. Growth requires proliferation of specified chondrocytes within the reserve zone, to produce longitudinal columns of chondrocytes (Kronenberg, 2003). The known master transcriptional regulators of chondrogenesis (*Sox9*) and osteogenesis (*Runx2*) are involved. Mutations in *SOX9* are associated with campomelic dysplasia, a perinatal lethal condition characterized by severe growth plate abnormalities, with short stature, bending of long bones, and kyphoscoliosis (Foster et al., 1994; Wagner et al., 1994). This is related to its role in the transactivation of genes necessary to initiate the chondrogenesis program in embryonic development, in conjunction with SOX5 and SOX6 (Akiyama et al., 2002; Lefebvre et al., 2019). Postnatally, SOX9 is also needed to prevent growth plate closure, as a gate keeper safeguarding the fate of the chondrocyte lineage (Haseeb et al., 2021). In the progression to hypertrophy, SOX9 is needed and RUNX2 alone is insufficient to induce chondrocyte hypertrophy (Dy et al., 2012), yet a coordinated down-regulation of *Sox9* (Wang et al., 2017; Zuo et al., 2018) and up-regulation of *Runx2* is required for mature chondrocytes to proceed towards ossification (Inada et al., 1999).

Pre-hypertrophic chondrocytes are formed at the onset of hypertrophy. They express Indian Hedgehog (*Ihh*) and the receptor for PTHrP (*Ppr/Pth1r*), which are key to the IHH/PTHrP feedback loop that regulates chondrocyte proliferation and hypertrophy (Karaplis et al., 1994; Vortkamp et al., 1996; St-Jacques et al., 1999). IHH acting as a morphogen has a positive effect on the proliferating chondrocytes. IHH also exert a long range effect activating *PTHrP* on chondrocytes closer to the periarticular region. PTHrP feeds back to receiving prehypertrophic chondrocytes that express the receptor, with a negative effect on hypertrophy (Kobayashi et al., 2002; Mak et al., 2008; van Donkelaar and Huiskes, 2007).

Interestingly, PTHrP-expressing (PTHrP^+^) cells in the reserve zone have properties of skeletal stem cells (SSCs) (Mizuhashi et al., 2018). Using Pthrp-creER;R26R^Confetti^ as a conditional tool to track the lineage of PTHrP^+^ cells in the reserve zone at P6, columns of clonal chondrocytes of fewer than 10 cells can be identified following 6 days of chase after tamoxifen injection: these columns expanded to more than 10 cells by 12 days of chase (Mizuhashi et al., 2018). PTHrP^+^ SSCs express a distinct set of immunology and cancer (CD) markers. Similar SSCs were identified in growth plates of foetal and neonatal periods in Col2-creERT:R26R^Confetti^ mice. These SSCs have self-renewal ability, reside in a stem cell niche that undergoes a switch into clonality, and with proliferation, forming the columns of chondrocytes (Newton et al., 2019). It is likely that the SSCs identified in these two studies overlap, and that expression of PTHrP regulated by IHH produced by pre-hypertrophic chondrocytes has a positive effect on chondrocyte proliferation in the reserve zone. The mammalian target of rapamycin complex 1 (mTORC1) might also be involved (Newton et al., 2018; Yan et al., 2016).

## 6 Towards chondrocyte hypertrophy

Chondrocyte hypertrophy occurs within the columns of proliferative chondrocytes. Cell expansion in the columns is considered as the second phase of bone growth following cell proliferation (Hunziker and Schenk, 1989), and contributes significantly to the linear growth (Hunziker et al., 1987). This is achieved through an almost 10-fold increase in water content, concomitant with an increase in cellular matrix and the number of various organelles such as the endoplasmic reticulum, Golgi apparatus, and mitochondria (Buckwalter et al., 1986), suggesting a switch in metabolic activity and energy flux. Rapid synthesis of membrane lipids and the cellular matrix would be needed to support this huge increase in cell size. Actin organization controls the chondrocyte phenotype and hypertrophy. An actin-binding gelsolin-like protein, Adseverin or Scinderin (SCIN) is highly expressed in hypertrophic chondrocytes, inducing a rearrangement of the actin cytoskeleton needed for the enlargement (Nurminsky et al., 2007). Indeed, an over-expression in chondrocytes in a 3D collagen gel culture induces a 3.5-fold increase in cell volume, together with expression of genes associated with hypertrophy, such as *Ihh* and *Col10a1* (Nurminsky et al., 2007).

A study using diffraction phrase microscopy that allows an estimation of cellular dry mass compared hypertrophic differentiation between fast (proximal tibia) and slow (radius) growing bones in the mouse, as well as in the fast-growing metatarsals of the Egyptian Jerboa (Cooper et al., 2013). Mammalian chondrocytes were shown to occur in three phases. First, cell volume and dry mass increase simultaneously; second, cells swell without significant increase in dry mass; third, there is again a coordinated increase in both cell volume and dry mass. The authors concluded that slow- and fast-growing bones differ in phase II, and most significantly in phase III, contributing to the different growth rate (Cooper et al., 2013).

In addition to cellular changes, drastic changes also occur in the extracellular environment. In particular, the cell switch from expressing the filbrillar type II collagen to the short-chain type X collagen (Yamasaki et al., 2001). Chondrocytes exist in chondrons, a term originally used to denote the pericellular matrix environment (Benninghoff, 1925; Poole, 1997). While the matrix in the territorial and inter-territorial regions between cells contain typical structural ECM proteins of hyaline cartilage such as type II, IX and XI collagens, and aggrecan (Eyre, 2001), the pericellular matrix of the chondrocytes is rich in type VI collagen, hyaluronan, fibronectin and laminin (Poole, 1997). Therefore, each chondrocyte is surrounded by a complex pericellular microenvironment, thought to serve as a micromechanical unit with the swelling pressure produced from the pericellular concentrations of hyaluronan and aggrecan to resist mechanical loading.

Chondrons when compacted vertically, deform laterally and shear under load, but recover completely when unloaded (Broom and Myers, 1980), acting as a protective unit, allowing limited cellular deformation and recovery. This property of chondrons might accommodate the initial increase in cell size in phase I of chondrocyte hypertrophy, but rapid changes may be needed to remodel the pericellular matrix to allow phase II and III to proceed. The change in collagens from type II to type X may allow for a more open ECM configuration and, with a higher content of hyaluronan (Farnum et al., 1984), pave the ECM space to complete hypertrophy. This notion would be consistent with the existence of columns of proliferative and hypertrophic chondrocytes as a micromechanical unit of a multicellular chondron. In the proliferative zone, each cell within the column is in direct contact and shares pericellular matrix with neighboring cells in the vertical orientation, but each column is separated by territorial/inter-territorial matrix in the horizontal direction. In the hypertrophic chondrocyte columns, the territorial/inter-territorial matrix is significantly smaller, allowing expansion in cell size (Fig. 1). The direction of growth and bone formation is from the erosion of hypertrophic cartilage and bone synthesis at the chondro-osseous junction. How this is coordinated with the induction of hypertrophy is unclear, as is whether a gradient of hypertrophic chondrocytes exists at different phases within one column of cells.

### 6.1 Molecular signals regulating chondrocyte hypertrophy

The down-regulation of *Sox9* expression and the up-regulation of *Runx2* expression signal the entry of the proliferative chondrocytes into hypertrophy (Ding et al., 2012). The proliferative chondrocytes exit the cell cycle and enter the G0 phase, as demonstrated by the expression activity of cyclin-dependent kinase inhibitor, p57^Kip2^ (Stewart et al., 2004). A transcription factor, C/EBPβ transactivates p57^Kip2^, promoting the transition to hypertrophic chondrocytes (Hirata et al., 2009). The rate and extent of chondrocyte proliferation is in part regulated by the suppression of p57^Kip2^ by PTHrP (MacLean et al., 2004).

**Fig. 2:**
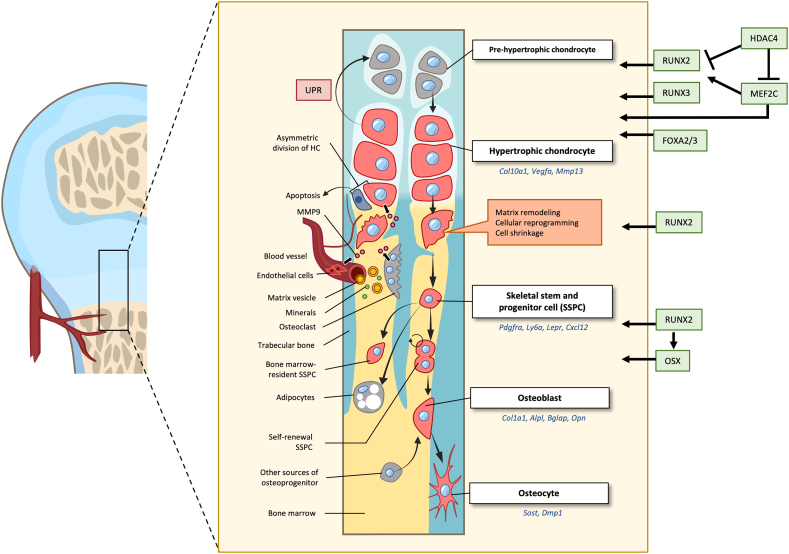
Current understanding of the molecular control and fate of hypertrophic chondrocytes in the developing growth plate Schematic diagram illustrating the molecular control and cellular changes focusing on the role and fate of hypertrophic chondrocytes in the process of endochondral ossification. The importance of Runx2 as a transcriptional factor functioning at all stages, from the initiation of chondrocyte hypertrophy to transition to an osteoblast. The plasticity of hypertrophic chondrocytes is highlighted with re-differentiation under cellular stress signals such as the unfolded protein response (UPR). The concept of asymmetric cell division for both cell shrinkage and apoptosis is included, as well as the proposed transition of a hypertrophic chondrocyte to a skeletal stem and progenitor cell (SSPC), with self-renewal ability and differentiation to osteoblast or adipocyte. The role of matrix remodeling at the chondro-osseous junction is also highlighted where the cross talk between hypertrophic chondrocytes and the invading blood vessels brings in essential ingredients (eg. calcium, matrix vesicles, and MMP9 which is supplied by endothelial cells, osteoclasts and hypertrophic chondrocytes). This interplay ensures a controlled conversion of the hypertrophic cartilage to bone, wherein osteoblasts can be derived from hypertrophic chondrocytes and other sources such as the pericytes from the blood vessel, and mesenchymal cells in the bone marrow.

Heterozygous mutations in *RUNX2* lead to cleidocranial dysplasia with abnormal bone development (Komori et al., 1997; Komori, 2022; Otto et al., 1997). *Runx2/3* are expressed in prehypertrophic and hypertrophic chondrocytes (Inada et al., 1999; Kim et al., 1999; Sato et al., 2008) (Fig. 2). RUNX2-regulated expression of *Ihh* (Yoshida et al., 2004), and inactivation of *Runx2* or reduced RUNX2 concentration due to expression of a dominant-negative *Runx2* allele leads to reduced chondrocyte hypertrophy (Inada et al., 1999). Inactivation of both *Runx2/3* leads to a more severe outcome, with a failure of chondrocyte hypertrophy and reduced *Col10a1* expression (Yoshida et al., 2004). On the other hand, over-expression of *Runx2* in chondrocytes results in premature chondrocyte hypertrophy in vitro (Enomoto et al., 2000) and in vivo (Takeda et al., 2001), marked by the expression of *Col10a1*. Indeed, RUNX2 promotes the expression of *Col10a1* (Drissi et al., 2003; Zheng et al., 2003).

In skeletal development, expression of *Runx2* is tightly regulated at many levels, that differ at different locations along the proximodistal axis in the developing limb. For example, SHOX2 is involved in the patterning proximal limb elements, and presents as a stylopod-specific regulator of *Runx2* expression in mice (Cobb et al., 2006), via the control of BMP4 (Yu et al., 2007) in regulating chondrocyte hypertrophy. DLX5 has also been shown as a positive regulator of chondrocyte hypertrophy. Retroviral misexpression in skeletal elements of developing chick limbs leads to the expansion of hypertrophic zone (Ferrari and Kosher, 2002). Conversely, inactivation of *Dlx5* in mice delayed chondrocyte maturation, and more chondrocytes in the proliferative zone (Bendall et al., 2003; Chin et al., 2007). DLX5 binds specifically to the distal (P1) promoter of *Runx2*, activating the expression of the type II isoform (Lee et al., 2005). *Dlx5* expression is under the regulation of BMP2 signaling (Lee et al., 2003), and its action on the P1 promoter is antagonized by MSX2 (Lee et al., 2005).

PTHrP signals through Gsα, an activator of cyclic AMP (cAMP) and protein kinase A (PKA) signaling, as a mediator in the inhibition of IHH signaling (Sakamoto et al., 2005; Jiang and Hui, 2008), as part of the negative feedback loop regulating hypertrophy. WNT signaling up-regulates the expression of *Ihh* (Mak et al., 2008). However, IHH signaling may also be upstream of WNT in this context, as *Wnt7a* and *Tcf1*, indicators of WNT signaling, are reduced in *Ihh*-deficient mice (Hu et al., 2005), forming a positive loop driving hypertrophy. WNT signaling has been shown to antagonize the action of PTHrP by independently promoting hypertrophy of the proliferating chondrocytes (Guo et al., 2009). Non-canonical WNT signaling through Wnt5a and Wnt5b also regulates the transition to hypertrophy, but independently of the IHH-PTHrP negative feedback loop. Wnt5a and Wnt5b appear to coordinate chondrocyte proliferation and differentiation by differentially regulating cyclin D1 and p130 expression, as well as *Col2a1* expression in chondrocytes (Yang et al., 2003).

Targets of PTHrP signaling include Zfp521 and Nkx3.2. Zfp521 is a zinc finger transcriptional coregulator expressed in prehypertrophic chondrocytes (Correa et al., 2010). Within the growth plate, Zfp521 associated with RUNX2, antagonizing its activity via an HDAC4-dependent mechanism. Thus, its inactivation leads to reduced chondrocyte proliferation because of early hypertrophic transition (Correa et al., 2010). *Nkx3.2* is expressed in the proliferative chondrocytes acting as a transcriptional repressor of *Runx2*, with a negative impact on hypertrophy (Lengner et al., 2005; Provot et al., 2006). Together, functioning through PTHrP signaling, Zfp521 and Nkx3.2 contribute to the negative feedback mechanism regulating the onset of hypertrophy.

BMP2 and 4 are expressed by prehypertrophic and hypertrophic chondrocytes (Nilsson et al., 2007). BMP signaling can induce chondrocyte hypertrophy in ex vivo limb culture (De Luca et al., 2001). It has been shown to interact directly with IHH and PTHrP pathways: for example, BMP2 induces the expression of *Ptch1*, the receptor for hedgehog signaling (Long et al., 2004), and BMP signaling potentiates or fine tunes the IHH-PTHrP negative feed-back loop (Minina et al., 2001). Further, BMP signaling can induce *Runx2* expression (Lee et al., 2003; Phimphilai et al., 2006) and PTHrP down-regulates the concentration of RUNX2 via induction of cyclin-D1-dependent phosphorylation of RUNX2/3, targeting them for proteasomal degradation (Zhang et al., 2009).

Notch signaling indirectly promotes chondrocyte hypertrophy via regulation of BMP signaling and cell cycle arrest (Shang et al., 2016). At the transcriptional level, Notch signaling mediated by HES and HEY factors have been shown to regulate the onset of chondrocyte hypertrophy via suppression of *Sox9* and *Col2a1* in development (Grogan et al., 2008; Hosaka et al., 2013; Chen et al., 2013; Kohn et al., 2015; Rutkowski et al., 2016) and osteoarthritis (Sugita et al., 2015; Hosaka et al., 2013). Suppression of *Sox9* was shown to be Rbpj-dependent notch signaling (Chen et al., 2013), likely via HES5, and together with HES1 that regulates *Col2a1* and *Acan* (Grogan et al., 2008) suppress chondrogenesis and promote chondrocyte hypertrophy (Rutkowski et al., 2016). In cultured chondrocytes, HES1 induced *Adamts5* and *Mmp13* (Sugita et al., 2015). Other targets of HES1 include Il6 (Sugita et al., 2015), and Il6 mediates suppression of *Acan* and induction of *Mmp13* expression by Notch in chondrocytes (Zanotti and Canalis, 2013), reinforcing the catabolism of the calcified hypertrophic cartilage at the chondro-osseous junction. Thus, together, these signals are likely to function co-ordinately at the earlier phase of hypertrophy.

On the other hand, the IGF1-IGF1R pathway is the key regulator of the later phase of chondrocyte hypertrophy (Cooper et al., 2013). In *Igf1* haploinsufficient mice, the height of the hypertrophic zone is specifically reduced (Wang et al., 1999), as well as the downstream pathway, PI3K-Akt, a major regulator of chondrocyte hypertrophy (Peng et al., 2003; Rokutanda et al., 2009; Ulici et al., 2009; Ulici et al., 2008). Indeed, IGF1 is a major hormone required for skeletal growth (Yakar et al., 2018), and is used clinically to treat children with skeletal growth abnormalities (Giustina et al., 2008).

Mice lacking Foxa2 and Foxa3 exhibit severe impairment of chondrocyte hypertrophy, with reduced expression of type X collagen, MMP13 and alkaline phosphatase (Ionescu et al., 2012). FOXA2 binds to conserved motifs in the Col10a1 promoter and can transactivate a Col10a1-luciferase reporter in chondrocytes. It is possible that FOXA2, together with other transcription factors such as RUNX2 and MEF2C, promote the expression of hypertrophic genes (Ionescu et al., 2012) (Fig. 2). Interestingly, epigenetic regulators also contribute to the regulation of chondrocyte hypertrophy. Histone deacetylase 4 (HDAC4) inhibits hypertrophy by suppressing RUNX2 and MEF2C (Vega et al., 2004; Arnold et al., 2007). HDAC4 activity is controlled by the balance between its cytoplasmic and nuclear localization. PTHrP promotes nuclear localization of HDAC4, mediated through dephosphorylation at residue S246 by phosphatase PP2A (Kozhemyakina et al., 2009), while the action of salt-inducible kinase 3 (SIK3) keeps HDAC4 in the cytoplasm (Sasagawa et al., 2012). Further, HDAC3 was shown to regulate chondrocyte hypertrophy through inhibition of phosphatase leucine-rich repeated phosphatase (PHLPP1), which in turn suppresses Akt signaling (Bradley et al., 2013). Thus, HDAC4 may function at an earlier stage of hypertrophy and HDAC3 at a later phase.

### 6.2 Metabolic and environmental dynamics of hypertrophic chondrocytes

An oxygen gradient exists in the avascular growth plate. The oxygen tension, partial pressure (pO2), of the proliferative zone is estimated to be 2–5 %, whereas that in the hypertrophic zone is estimated to be 0.5–1.0 % (Shapiro et al., 1997). Despite being located closer to the vasculature at the ossification front, hypertrophic chondrocytes experience a lower oxemic state. This might be due to their enlarged size: a lower surface-area-to-volume ratio might impede the intracellular transport of oxygen. As a result, the upper hypertrophic chondrocytes often exhibit stronger hypoxic signals, as detected by nitroimidazole EF5 and hypoxyprobe (Bentovim et al., 2012; Schipani et al., 2001). An early study using ^31^P NMR to assess ATP synthesis and hydrolysis kinetics showed resting and hypertrophic chondrocytes use both glycolysis and mitochondrial respiration for energy production, but glycolysis dominates. Further, hypertrophic cells rely more on mitochondrial respiration than the resting chondrocytes (Pollesello et al., 1991).

HIF-1α is a key transcription factor in the induction of a hypoxic response. In the presence of oxygen, HIF-1α is hydroxylated by pyruvate dehydrogenase (PHD), and the hydroxylated HIF1α is recognized by the E3 ubiquitin ligase, pVHL, leading to proteasomal degradation. When oxygen tension is low, HIF-1α is stabilized and translocated to the nucleus, where it influences the expression of around 150 genes that are either up or down regulated (Greijer et al., 2005), which are mostly involved in energy metabolism or angiogenesis. HIF-1α-mediated hypoxic response down-regulates the tricarboxylic acid cycle to reduce oxygen consumption (Bentovim et al., 2012), allowing the central chondrocytes to survive in a hypoxic milieu (Schipani et al., 2001), and to process and secret ECM proteins from the endoplasmic reticulum (Bentovim et al., 2012; Stegen et al., 2019). A major downstream target of HIF-1α is VEGF, which regulates angiogenesis (Semenza, 2012). Overexpression of VEGF in growth plate chondrocytes was not sufficient to prevent cell death in the HIF-1α-deficient growth plate (Maes et al., 2012). Given that VEGF is not solely regulated by HIF-1α, it appears that they function together as key regulators, balancing availability and the handling of oxygen, while HIF-1α protects chondrocytes from excessive hypoxia, limiting the need for oxygen consumption (Maes et al., 2012).

HIF-1α promotes a metabolic switch towards glycolysis via upregulating a number of glycolytic enzymes, including phosphoglycerate kinase (PGK) and lactate dehydrogenase (LDHA), leading to an increase in the concentrations of pyruvate and lactate (Pfander et al., 2004; Schipani et al., 2001; Chen et al., 2022). Interestingly, co-treatment of human chondrocytes with lactate and NaF, an oxidative phosphorylation inhibitor, can induce hypertrophy in vitro (Nishida et al., 2013). In vivo, a conditional deletion (*Aggrecan-cre–ERT2*) of *Ldha* in chondrocytes reduced the number of hypertrophic chondrocytes in articular cartilage after OA induction by meniscal-ligamentous injury (Arra et al., 2020). This suggests lactate can drive chondrocyte hypertrophy. However, in the OA model, the switch in metabolic status is thought to be driven by the presence of inflammation and reactive oxygen species (ROS). Whether the developmental and pathological pathways to hypertrophy share a similar metabolic switch to glycolysis is not clear, but both hypoxia and ROS could induce similar downstream stress signals, such as the unfolded protein response (UPR) (Hetz et al., 2020) or the integrated stress response (ISR) (Pakos-Zebrucka et al., 2016; Costa-Mattioli and Walter, 2020). Further, while ER stress is known to be a contributing factor in skeletal dysplasia (Bateman et al., 2009; Boot-Handford and Briggs, 2010; Tsang et al., 2010), ER stress in hypoxic chondrocytes also plays a role in normal skeletal development (Patra et al., 2007; Saito et al., 2009), supporting possible converging role as a contributing factor to hypertrophy.

An increasing concentration of lactate may lead to progressive stabilization of HIF-1α, as demonstrated in a study of adipocyte differentiation (Feng et al., 2022). A similar situation might occur in chondrocytes. Feng et al. (2022) showed that lactate can directly bind and inhibit PHD2, thus preventing HIF-1α degradation. Indeed, stabilization of HIF-1α in cartilage leads to ectopic cellular enlargement throughout the epiphyseal cartilage anlagen, including the reserve and proliferative zones (Pfander et al., 2004; Chen et al., 2022), coupled with enhanced expression of *Col10a1*, *Mmp13* and *Ihh,* all markers of chondrocyte hypertrophy (Chen et al., 2022; Cheng et al., 2016). Further, inactivation of *HIF-1α* leads to a loss of *p57*^*Kip2*^ that is required for proliferating chondrocytes to exit the cell cycle and enter hypertrophy (Schipani et al., 2001). Conversely, stabilization of HIF-1α results in reduced proliferation of chondrocytes (Chen et al., 2022; Pfander et al., 2004; Stegen et al., 2019). Thus, HIF-1α is necessary for the initiation of hypertrophy. HIF-1α also regulates ECM production in the cartilage growth plate, as it regulates the expression of cP4H, an enzyme that catalyses hydroxylation of collagens in hypoxic growth plate cartilage (Bentovim et al., 2012), in particular, the proline residues essential for stabilization of the triple helical molecules and subsequent secretion from cells (Berg and Prockop, 1973). Thus, HIF-1α would be needed for the production of the various collagens in the different zones.

## 7 Hypertrophic chondrocytes and vascularization at the chondro-osseous junction

The presence of hypertrophic chondrocytes is linked to the initiation of cartilage mineralization and vascular invasion from the calcification front, leading to the conversion to bony trabeculae. This is a sequential erosion process, not a migration of the hypertrophic chondrocytes towards the calcification front (Hunziker, 1994). It is essentially a coordinated remodeling process, whereby the hypertrophic chondrocytes at the chondro-osseous junction are responding to the changing environment from the calcification front (Ma et al., 2020), with three integrated events, vascularization, mineralization and ECM remodeling.

VEGF, a potent angiogenic factor, is produced by hypertrophic chondrocytes (Gerber et al., 1999). Invasion of blood vessels into cartilage is associated with tissue-specific expression of VEGF in the growth plate. Conditional inactivation of *Vegf* in *Col2a1*-expressing chondrocytes in mice impairs vascular invasion, leading to increased death of hypertrophic chondrocytes (Zelzer et al., 2004). Similar changes in the growth plate are seen when VEGF is sequestered by its antagonist, Flt-(1–3)-IgG (Gerber et al., 1999). *Vegf* expression is associated with the expression of *Runx2* (Zelzer et al., 2001). Over-expression of *Runx2* in chondrocytes under a *Col2a1* promoter enhanced chondrocyte hypertrophy and expression of *Vegf* (Takeda et al., 2001). Such studies support the notion that *Runx2* is a multifunctional transcription factor in skeletal development, involved in coordinating the sequential phases of endochondral ossification, as well as the transdifferentiation to osteoblasts (Qin et al., 2020). However, when *Runx2* was conditionally inactivated in hypertrophic chondrocytes using *Col10a1-Cre/Runx2*^*flox/flox*^ mice, *Vegf* was not expressed but vascularization at this site was not significantly impaired: enhanced *Vegf* expression by osteoblasts in the bone collar was observed as a possible compensation (Qin et al., 2020). Thus, VEGF from multiple sources can contribute to vascular invasion of the cartilage.

## 8 Hypertrophic chondrocytes and biomineralization

Biomineralization requires a source of ions, and enzymes to facilitate and regulate the process. To provide such an environment, hypertrophic chondrocytes produce the ECM and coordinate the deposition of hydroxyapatite (HA) to the ECM. Transmission electron microscopy showed high exocytotic activity in the plasma membrane of the hypertrophic chondrocytes, with budding vesicles proposed to be a source of matrix vesicles (Anderson, 1969; Cecil and Clarke Anderson, 1978; Akisaka and Gay, 1985; Buckwalter et al., 1987). Mineralizing matrix vesicles are membrane bound particles of cellular origin, typically containing proteins and lipids that chelate P_i_ and Ca^2+^. There is a graded distribution of matrix vesicles along the hypertrophic zone with varying mineralizing competency. Vesicles with higher alkaline phosphatase activity and that contain more P_i_ and Ca^2+^ are more competent (Boyan et al., 1988; Kirsch et al., 1997; Lin et al., 2018).

While some studies showed hypertrophic chondrocytes can be a source of matrix vesicles, a study of a 9-week-old mouse growth plate suggested a significant contribution from the blood vessels (Haimov et al., 2020). In fact, this study did not find evidence of hypertrophic chondrocytes containing organelles with minerals that resemble matrix vesicles or export of mineral to the surrounding matrix. However, intracellular mineral particles were located within cells juxtaposed to the blood vessels, likely to be osteoblasts (Haimov et al., 2020). Further, matrix vesicles were found present within blood vessels but not within cells in the blood vessels, consistent with an observation in chick embryos (Kerschnitzki et al., 2016), and the view that minerals for bone formation are supplied as ions in solution via the circulation (Neuman and Neuman, 1953).

The formation of bone in close contact with blood vessels is also consistent with this notion. Mineralization of cartilage and conversion to bone take place in close association with hypertrophic chondrocytes (Boyde and Jones, 1983) adjacent to blood vessels. Thus, an intimate relationship exists whereby minerals from the blood vessels are deposited onto the collagenous matrix produced by hypertrophic chondrocytes/osteoblasts, facilitated by the presence of matrix vesicles (Morris et al., 1983). The discrepancy in whether hypertrophic chondrocytes act as a sources of matrix vesicles may be related to whether this is studied prenatally and postnatally, or due to species differences in the various studies, in which the mechanism may vary. The cellular regulation of HA formation in vivo is not well understood, partly because of the difficulty in visualizing the microscopic apatite. Nonetheless, morphological studies coupled with the spatial gene expression related HA formation at the hypertrophic zone have provided some insights into how the calcium crystal is first formed within the matrix vesicles prior to its release into the ECM (Cui et al., 2016).

Hypertrophic cartilage acts as a buffer zone between bone and cartilage, to suppress mineralization in the cartilaginous zones. In addition to various enzymes that regulate mineralization, hypertrophic chondrocytes express a vitamin K-dependent calcification inhibitor, Matrix Gla Protein (MGP) (Ikeda et al., 1992; Luo et al., 1997; Yagami et al., 1999). MGP protects cartilage from being annexed by calcification, and fine tunes the tempo of crystal nucleation at the hypertrophic zone in synchrony at the ossification front. This is consistent with its expression at the periphery of the developing SOC and the upper region of the growth plate (Dan et al., 2012; Ikeda et al., 1992; Luo et al., 1997). Thus, deletion of *Mpg* in mice resulted in ectopic mineralization of the cartilage including the growth plate, together with the calcification of several vascular tissues. This was accompanied by abnormal cellular organization in the growth plate, producing mice with short stature and reduced lifespan, characteristics similar to Singleton-Merten syndrome (Luo et al., 1997). In contrast, overexpression of *Mpg* in the developing chick limb inhibited cartilage mineralization with delayed chondrocyte maturation and endochondral ossification (Yagami et al., 1999). Type X collagen as a specific marker of hypertrophic chondrocytes has been implicated in regulating the distribution of matrix vesicles in the hypertrophic zone: in *Col10a1-null* mice, abnormal transposition of matrix vesicles and proteoglycan granules towards the proliferating zone was observed (Kwan et al., 1997), suggesting type X collagen negatively regulates mineralization through compartmentalization of matrix vesicles and ECM components.

Another inhibitor of ECM mineralization is extracellular inorganic pyrophosphate (PP_i_), produced when nucleotide pyrophosphatase/phospho-diesterase-1 (NPP1) catabolizes extracellular ATP (Meyer, 1984; Harmey et al., 2004; Hessle et al., 2002; Terkeltaub, 2001; Majeska and Wuthier, 1975). Intracellular PP_i_ is transported to the ECM via the channeling function of the ankylosis protein (ANK) (Ho et al., 2000). Within the mineralizing milieu, tissue-nonspecific alkaline phosphatase (TNAP) fine tunes the level of extracellular PP_i_, maintaining a permissive P_i_/PP_i_ ratio for proper bone mineralization (Johnson et al., 2000). While our understanding of the role of these regulators of mineralization is largely from in vitro studies of osteoblasts or global gene inactivation in mice, the mechanism is likely to be similar in hypertrophic cartilage where matrix vesicles and Akp2 are involved in HA crystal formation (Harmey et al., 2004).

## 9 Extracellular matrix remodeling of the hypertrophic cartilage

The hypertrophic cartilage is situated between the sites of two discrete and tightly coordinated matrix remodeling events. One involves the conversion of an avascular and nonmineralized cartilage matrix from the proliferative zone to a mineralized hypertrophy cartilage infiltrated with blood vessels. This is represented by the degradation of a type II collagen-containing ECM (Mwale et al., 2002), rich in proteoglycans, and its replacement with ECM produced by hypertrophic cartilage containing type X collagen and different proteoglycans. The second event is the conversion of the hypertrophic cartilage to bone, and a switch to a type I collagen-containing matrix with structural glycoproteins conducive for mineralization and establishment of the bony trabeculae.

The ECM provides not only the structural support to the growing physis, but also critical information to the cells within, such as mechanical signals and the signaling molecules entrapped within the ECM, with graded changes from the pericellular, to the territorial and the inter-territorial matrix (Wilson et al., 2012; Prein and Beier, 2019). The process of endochondral ossification is highly organized and balanced: progressive alterations in the composition and organization of the ECM accompany the cellular changes, and vice versa, such that the rate of bone formation matches the rate of chondrocyte proliferation and hypertrophy (Blair et al., 2002). This review will not address the ECM composition, but rather, the matrix metalloproteinases (MMPs), enzymes that are involved in ECM degradation (Ortega et al., 2004), in particular, the complex conversion of hypertrophic cartilage to bone.

The ECM produced by and surrounding the hypertrophic chondrocytes is in part degraded by the cells themselves at the chondro-osseous junction, as well as by osteoblasts that degrade the mineralized cartilage, and become permissive to vascularization and bone formation. Known key enzymes involved are MMP9, MMP13 (MMP1 in human) and MMP14 (MT1-MMP) (Ortega et al., 2004; Ortega et al., 2010; Stickens et al., 2004), produced by the hypertrophic chondrocytes. MMP9 and MMP10 also come from the endothelial cells of the invading capillaries (Vu et al., 1998). Given that type X collagen contains multiple MMP cleavage sites (Ninomiya et al., 1986), degradation of type X collagen is likely the initiation event, while the activity of the MMPs is further regulated by the presence of tissue inhibitors of MMPs (TIMPs 1–4).

Hypertrophic chondrocytes also express RANKL (Kishimoto et al., 2006; Xiong et al., 2011), a ligand that binds to RANK, its receptor that is present on the surface of preosteoclasts, regulating osteoclast maturation and activity. This intimate relationship ensures an orderly removal of the mineralized matrix. Osteoclasts distinguished by the expression of tartrate-resistant acid phosphatase (TRAP) provide additional MMPs (MMP9, 10 and 14), and produce the acidic microenvironment required for demineralization (Blair et al., 1989; Ortega et al., 2010; Vu et al., 1998; Bord et al., 1998). Thus, the conditional inactivation of *Rankl* using *Col10a1-Cre* or *Ocn-Cre* in mice leads to an enlarged hypertrophic zone because of reduced osteoclast activity at the ossification front (Xiong et al., 2011). MMP9 activity is concentrated proximal to the chondro-osseous junction, where vascular invasion occurs, and its role is reinforced by the three concerted sources of this enzyme (Vu et al., 1998; Lee et al., 1999) (Fig. 2). Inactivation of *Mmp9* in mice leads to a pronounced phenotype at the primary site of ossification but little alteration at the secondary site (Vu et al., 1998), indicating differences between these ossification centers and differential MMP requirements.

Inactivation of *Mmp13* in mice also results in a mildly expanded hypertrophic zone (Inada et al., 2004; Stickens et al., 2004), demonstrating its role produced by hypertrophic chondrocytes. Inactivation of MMP9 and MMP13 together caused greater expansion of the hypertrophic zone, accompanied by reduced breakdown of aggrecan (Stickens et al., 2004). Thus, MMP9 and MMP13 act together in primary and secondary ossification. MT1-MMP (MMP14) is a membrane-bound MMP (Sato et al., 1994). Unlike in *Mmp9-null* mice, inactivation of *Mt1-mmp* had little effect on the primary ossification, but more on secondary ossification (Zhou et al., 2000), supporting site-specific differences in MMP requirements. In summary, proteolytic activity of MMPs at the chondro-osseous junction not only remodels the ECM but also indirectly assists in the recruitment of different cell types, communication, and function, primed by the expression of *Runx2*, that regulates the expression of *Vegf* and *Mmp13*.

## 10 Plasticity of chondrocytes and hypertrophic chondrocyte

The lineage progression of chondrocyte differentiation in endochondral ossification signifies a unidirectional process under the control of the in vivo environment in normal development. As for many tissues and organs, maintaining the cellular phenotype in vitro is very difficult, particularly so for chondrocytes. Chondrocytes released from their endogenous environment by enzymatic digestion and cultured in a 2D monolayer condition will quickly dedifferentiate, with morphological and gene expression changes (Benya et al., 1978). The dedifferentiation is progressive and happens quickly within 2–3 passages (Lefebvre et al., 1990). The initial changes are upregulation of the expression of fibroblastic genes (type I collagen and versican) with proliferation. Subsequent passaging/expansion leads to downregulation of expression of chondrocyte genes (type II collagen and aggrecan) (Von Der Mark et al., 1977; Lefebvre et al., 1990; Lin et al., 2008).

Extensive passaging reduces the potential to be re-differentiated (Kang et al., 2007). *Re*-differentiation to a more chondrogenic phenotype can be achieved by culturing the dedifferentiated cells in a 3D environment, in agarose (Benya and Shaffer, 1982) or alginate beads (Lemare et al., 1998). Further, the chondrogenic phenotype of primary chondrocytes can be maintained better as pellet cultures (Yeung et al., 2019), suggesting cell shape is an important characteristic. However, treatment of dedifferentiated chondrocytes with dihydrocytochalasin B can lead to re-expression of chondrocyte genes without much change in cell shape (Benya et al., 1988), suggesting signals associated with microfilament cytoskeletal structure are part of the regulatory mechanism, perhaps proximal to changes in cell shape.

Indeed, an interplay between cytoskeletal polymerization and the chondrogenic phenotype in chondrocytes passaged in monolayer culture has been demonstrated (Parreno et al., 2017). This study showed that actin polymerization status regulates chondrocyte dedifferentiation, and reorganization of the cytoskeleton by actin depolymerization may be an active redifferentiation regulatory mechanism. The capacity for dedifferentiation and redifferentiation clearly show plasticity characteristics of chondrocytes. For redifferentiation, some intrinsic chondrogenic signals are gradually lost with population doubling, established to be around 4 doublings (Giovannini et al., 2010). On the other hand, chondrocytes can be efficiently expanded and induced to have mesenchymal stem/stromal cell (MSC) characteristics by using supplements such as FGF2, and the resultant dedifferentiated cells are able to direct ectopic cartilage and bone formation in BALB/c nude mice (Lee et al., 2017). However, when dedifferentiated chondrocytes were generated in MSC culture medium, there was a preference for redifferentiation to cartilage and not bone (Lee et al., 2017). Thus, it is possible to modify and direct the dedifferentiation process through addition of growth factors and/or choice of culture medium.

Dedifferentiation of hypertrophic chondrocytes has not been studied in the same in vitro context. The plasticity of hypertrophic chondrocytes was demonstrated in mouse models of MCDS (Warman et al., 1993) and reviewed in (Chan and Jacenko, 1998; Bateman et al., 2005). Almost all the mutations in these models are in the NC1 domain of type X collagen, affecting trimer assembly (Chan et al., 1996; Chan et al., 1995) or cleavage of the signal peptide (Chan et al., 2001), and severely impairing secretion from the endoplasmic reticulum. However, the primary molecular consequence appears not to be due to an absence or reduced concentration of type X collagen in hypertrophic cartilage, consistent with there being little or no observable phenotype in mice after inactivation of *Col10a1* (Kwan et al., 1997; Rosati et al., 1994). However, transgenic (Ho et al., 2007; Tsang et al., 2007) or gene targeted (Rajpar et al., 2009; Forouhan et al., 2018) mice with equivalent human mutations in the mouse *Col10a1* gene recapitulated the human MCDS characteristics with short limbs and *coxa vara*. Thus, the molecular consequence is not a loss-of-function, but a gain-of-function from misfolded type X collagen chains activating the UPR (Tsang et al., 2007).

The cellular outcome observed in the MCDS mouse models is due to redifferentiation or reprogramming of the hypertrophic chondrocytes to a more immature state, closer to pre-hypertrophic or proliferative chondrocytes, interrupting endochondral ossification, resulting in reduced bone growth and dwarfism (Tsang et al., 2007) (Fig. 2). At the molecular level, the reprogramming is associated with the PERK/ATF4 axis of the UPR, where ATF4 can transactivate the expression of *Sox9* in hypertrophic chondrocytes (Wang et al., 2018). The involvement of the UPR and ATF4 was confirmed when pharmacological treatments with inhibitors of the UPR (Wang et al., 2018) and enhanced removal of the misfolded proteins using carbamazepine (Mullan et al., 2017; Forouhan et al., 2018) partially restored endochondral ossification and bone growth. This ability of chondrocytes to transition between prehypertrophic and hypertrophic states implies an intrinsic plasticity. The degree of reprogramming of hypertrophic chondrocytes in MCDS mice is dosage dependent, reflected in different levels of UPR activation in mice heterozygous or homozygous for the mutant alleles (Kung et al., 2012). Indeed, hypertrophic chondrocytes have some capacity to cope with ER stress without triggering the UPR (Kung et al., 2012), which is consistent with the need for survival in a harsh hypoxic environment, and the general integration of the cellular stress pathways via the integrated stress response (Costa-Mattioli and Walter, 2020; Pakos-Zebrucka et al., 2016).

The precise mechanism by which cellular stress pathways contribute to the intrinsic plasticity of hypertrophic chondrocytes remains to be determined. There are indications that other cellular surveillance mechanisms may be involved. Most *COL10A1* mutations that cause MCDS are associated with aspects of protein misfolding and stability of the triple helix, with the potential of activating the UPR. However, an autosomal recessive chondrodysplasia with severe short stature caused by a c.133C > t (p.pro45Ser) mutation in the NC2 domain of *COL10A1* was reported for a large consanguineous family (Ain et al., 2018). Heterozygous carriers exhibited a mild form of dwarfism, while homozygous individuals had severe skeletal dysplasia and marked lower limb deformities. Based on the known biochemistry of type X collagen biosynthesis, this mutation would not be expected to interfere with intracellular processing and secretion of the mutant molecule. How this might be associated with reprogramming of hypertrophic chondrocytes is unclear. Further, nonsense mutations in *COL10A1* have been shown to activate nonsense-mediated mRNA decay (NMD), by which the mutant mRNA is preferentially degraded (Tan et al., 2008; Bateman et al., 2003).

NMD is a mechanism by which cells survey and control the quality of mRNAs. Mutated mRNAs are actively degraded to protect the integrity of the transcriptome (Kurosaki et al., 2019). In MCDS patients heterozygous for a p.Y632X mutation in *COL10A1*, mutant mRNAs were not detected in the growth plate cartilage (Chan et al., 1998), suggesting complete degradation by NMD. While this leads to haploinsufficiency for type X collagen, the disease mechanism is likely linked to NMD. Interestingly, distinct cross talk exists between the cellular stress pathways and the ISR that modulates the NMD (Kurosaki et al., 2019). For example, activation of the PERK sensor leads to the phosphorylation of eIF2α and suppression of NMD. NMD suppression enables the expression of NMD targets, including ATF4, CHOP and ATF3, which alleviate cellular stress. Thus, active UPR/ISR and NMD could play a role in hypertrophic chondrocyte plasticity. Of note, NMD was recently shown to be a potential modulator of malignancy in cancer biology (Tan et al., 2022).

## 11 Hypertrophic chondrocytes can transit to become osteoblasts and adipocytes

The fate of hypertrophic chondrocytes has long been debated. While the notion that hypertrophic chondrocytes undergo apoptosis in endochondral ossification, and the hypertrophic cartilage is replaced by bone has dominated the field (Anderson and Parker, 1966; Bentley and Greer, 1970; Hanaoka, 1976; Hunziker et al., 1984; Farnum and Wilsman, 1989), the opposing theory is that hypertrophic chondrocytes can transdifferentiate to osteoblasts (Crelin and Koch, 1967; Lutfi, 1971; Aghajanian et al., 2017; Moskalewski and Malejczyk, 1989; Shimomura et al., 1975; Kahn and Simmons, 1977; Yoshioka and Yagi, 1988; Galotto et al., 1994). These theories are primarily based on morphological studies of in vivo and ex vivo chondrocytes and hypertrophic chondrocytes in mammalian and avian growth plate cartilage. A mechanism that reconciles both theories was proposed, by which osteogenic differentiation of hypertrophic chondrocytes involves asymmetric cell division, where one daughter cell transdifferentiates to an osteoblast, while the other undergoes apoptosis (Roach et al., 1995) (Fig. 2). This study in femur growth plate of 14-day-old chick embryos in organ cultures was based on the electron micrograph finding of two cells with different morphologies in the one lacuna, with one cell showing evidence of active protein synthesis containing many ribosomes and rough endoplasmic reticulum, while the other appeared to have disintegrated (Roach et al., 1995).

While apoptosis of hypertrophic chondrocytes does occur in endochondral ossification, recent studies showed that it is of relatively low prevalence. Compelling evidence for a transition to osteoblasts comes through studies using genetic tools in mice to mark hypertrophic chondrocytes and their descendants (*Col10a1-mCherry*, *Col10a1-GFP*), or inducible cell lineage tracing studies in mice of chondrocytes (*Col2a1-Cre™*; *Acan-Cre*^*ERT2*^) or hypertrophic chondrocytes (*BACCol10Cre*, *Col10a1*^*int2*^*-Cre*, *Col10a1-Cre*; *Col10a1-Cre*^*ERT2*^). Although in some of these *Cre* mice, expression in the desired cell types is imperfect due to the use of transgenes under the control of *Col2a1* and *Col10a1* promoters and regulatory elements, these studies together have provided strong support for a lineage of chondrocytes to hypertrophy, and then transition to osteoblasts (Yang et al., 2014a; Yang et al., 2014b; Zhou et al., 2014b), which has been reviewed (Hallett et al., 2021; Javaheri et al., 2018; Kodama et al., 2022; Tsang et al., 2015; Wang et al., 2022b). As *Col10a1* is specifically expressed by hypertrophic chondrocytes in the growth plate, lineage tracing using *Col10a1-Cre* mice provided the most direct evidence, and the transgenic *BacCol10Cre* (Gebhard et al., 2008) and the gene-targeted *Col10a1-CreERT2* (Yang et al., 2014b) mice provided the most specific expression of the *Cre* recombinase in hypertrophic chondrocytes (Yang et al., 2014b; Zhou et al., 2014b), as demonstrated when crossed with ROSA reporter mice.

Hypertrophic chondrocytes express many osteoblast genes, such as alkaline phosphatase (*Alpl*), osteocalcin (*Ocn/Bglap*) and osteopontin (*Opn/Bsp-I*), but not type I collagen (*Col1a1*) (Gauci et al., 2019; Miao et al., 2004), suggesting that they may be “primed” to become osteoblasts. A transition to *Col1a1*-expressing osteoblasts from *Acan*-expressing cells (*AcanCre*^*ERT2*^) (Henry et al., 2009) was demonstrated using an osteoblast reporter, *2.3* *kb Col1a1-GFP*: the descendants, *Rosa-Tomato* positive cells, expressed GFP, presumably via a hypertrophic chondrocyte intermediate (Zhou et al., 2014b). *Col1a1* expression was also identified in descendent cells marked in the *Col10a1-CreERT2/Rosa-LacZ* mice (Yang et al., 2014b). Indeed, these cells further matured to *Sclerostin* (*Sost*)-expressing osteocytes in trabecular and cortical bones (Yang et al., 2014b) at prenatal (E15.5-E18.5), early postnatal (P5–10) and adult (3 months) stages. The precise contribution of this osteoblast lineage to the primary spongiosa is not clear but is estimated to be around 10–30 % for endochondral ossification in the growth plate in early postnatal life in mice (Yang et al., 2014b; Park et al., 2015). The functional role of this chondrocyte transition lineage to osteoblasts also has not been studied, but differences exist in the expression level of osteogenic genes (*Runx2*, *Sp7*, *Bsp-II*) in relation to their origin (subchondral, trabecular and cortical), as well as VEGF-A in association with the vasculature (Shah et al., 2015; Clarkin and Olsen, 2010).

The transition mechanism and the molecular signals involved are also not clear. Given the important role of the transcription factor, *Runx2,* in chondrocyte hypertrophy and osteogenesis, it is not surprising that it also plays a key role in the transition process (Qin et al., 2020), as does the level of SOX9, in that inactivation of *Shp2* using *BacCol10a1-Cre* (Gebhard et al., 2008) leads to elevated and sustained level of SOX9 in hypertrophic chondrocytes, impairing the transition to osteoblasts (Wang et al., 2017) (Fig. 2). Both WNT and IHH signals are osteogenic and are likely to play a role. Inactivation of β-catenin using either *Acan-CreERT2* or *Col10a1-Cre* resulted in less “chondrocyte-derived” bone, whereas activation of β-catenin using *Acan-CreERT2* enhanced the transition to bone (Jing et al., 2018). IHH signaling mediates in part the activation of Runx2 and Osx via GlI2 (Shimoyama et al., 2007). A recent study showed that IHH signaling orchestrates the cartilage-to-bone transition independently of Smoothened (*Smo*) (Wang et al., 2022a). Inactivation of *Smo* in hypertrophic chondrocytes (*Col10a1-Cre*^*ERT2*^) had little effect on growth plate function, but inactivation of *Ptch1,* which normally suppressed SMO activity in the absence of IHH, disrupted the forming primary spongiosa with active proliferation of hypertrophic chondrocyte-derived osteoblasts, and impaired maturation to osteocytes. The details of how IHH-related signaling operates remains to be elucidated (Wang et al., 2022a), with possible interaction with systemic level of thyroid hormone through receptors, TRα1 and TRβ1 (Aghajanian et al., 2017).

The pathway by which a hypertrophic chondrocyte becomes an osteoblast is still unclear. It may not be a direct transdifferentiation but occur via dedifferentiation to an intermediate with stem/progenitor cell properties, which would require a significant reduction in cell size. How this could be achieved is uncertain. One possibility is via asymmetric cell division (Roach et al., 1995), and cytoskeletal changes observed in in vitro dedifferentiation (Rodríguez et al., 2004). Re-entry into the cell cycle could also contribute to this cell shrinkage (Hu et al., 2017). This study analyzed the cartilage to bone transformation during fracture healing, supported by the incorporation of BrdU and expression of *Ki-67* by hypertrophic chondrocytes at the transition zone. Pluripotent factors such as *Sox2*, *Oct4* and *Nanog* are activated in hypertrophic chondrocytes at this zone, consistent with a dedifferentiation or reprogramming event. Furthermore, there is a transition to a more osteogenic signature with the expression of *Col1a1* (Hu et al., 2017), consistent with similar findings in the growth plate and primary ossification center (Yang et al., 2014b; Park et al., 2015). Indeed, small *Col10a1+* (*BacCol10a1ERT2/Rosa-YFP*)-descendent cells expressing *Osx* with mitotic (BrdU incorporation) activity were identified by confocal microscopy, supporting the presence of transitory cells or chondrocyte-derived osteoprogenitor cells (Park et al., 2015). Isolated chondrocyte-derived osteoprogenitor cells are small (4-6 μm in diameter), express typical osteogenic (*Col1a1*, *Osx* and *Runx2*), and stem cell (*CD34*, *Sca1*, *Sox2*, and *c-Myc*) genes, and differentiate into osteoblasts in vitro (Park et al., 2015).

The fate of hypertrophic chondrocytes appears to go beyond endochondral ossification: they also provide marrow-associated skeletal stem and progenitor (SSPC) cells (Long et al., 2022). Single cell transcriptome analysis of prenatal and postnatal skeletal rudiments of Col10a1-Cre^ERT2^;Rosa26^fs-tdTomato^ and Col10a1-Cre;Rosa26^fs-tdTomato^ mice confirmed the presence of hypertrophic chondrocyte-derived osteoprogenitors expressing SP7 that had re-entered the cell cycle (Park et al., 2015), but also identified hypertrophic chondrocyte-derived SSPCs that were dually primed for both osteoblast and adipocyte differentiation in the marrow space (Long et al., 2022). The transition via a progenitor state is consistent with a finding in zebra fish where chondrocytes re-enter the cell cycle and express leptin receptor (*Lepr*) (Giovannone et al., 2019). These cells marked by *Sox10* and *Col2a1*, contributing to mesenchymal cells, osteoblasts, and marrow adipocytes within adult bone (Giovannone et al., 2019). Further, a study in mice showed that IRX3 and IRX5 are critical in the hypertrophic chondrocyte lineage decision to osteoblasts by inhibiting the adipogenic fate (Tan et al., 2020).

The characteristics of these SSPCs are similar to those of previously identified SSPCs: both express *Pdgfra*, *Ly6a*, *Lepr*, and *Cxcl12* (Chan et al., 2015; Zhou et al., 2014a; Ambrosi et al., 2017; Böhm et al., 2019). Together, these exciting findings provide new insights into the fate of hypertrophic chondrocytes, suggesting that these cells are not terminally differentiated but have continued roles in osteogenesis in both prenatal and postnatal life, and in skeletal homeostasis and repair in postnatal life (Fig. 2).

## 12 Conclusion

The dogma that hypertrophic chondrocytes are terminally differentiated cells destined to die has been challenged for decades. Much of what we know about hypertrophic chondrocytes comes from studies of rodent and avian skeletal development. Their unique spatial location within the cartilage growth plates and their lineage derived from proliferative chondrocytes through hypertrophy, with an expansion of almost 10× in cell volume, has puzzled researchers—what is the need for such an expansion, following the highly proliferative phase in endochondral ossification? The rapid change in the ECM composition has prompted the hypothesis that hypertrophic cartilage is a transition tissue between cartilage and bone, and prepares the cartilage for a conversion to bone. Indeed, hypertrophic cartilage serves this purpose together with invasion of vasculature.

This review summarized the current understanding of the role of hypertrophic chondrocytes in endochondral ossification. Studies using current molecular and genetic tools in mice support the century-long view of many researchers that hypertrophic chondrocytes are not terminally differentiated and could become bone cells, possibly through a transition process of dedifferentiation to a more progenitor-like cells and then redifferentiation to osteoblasts. While the precise role for this osteoblast lineage has yet to be delineated, it accounts for a significant fraction, varying between estimates of 15–80 % of the hypertrophic chondrocytes transitioning to osteoblasts in the primary spongiosa during prenatal, postnatal bone growth, and in fracture repair in mice (Long et al., 2022; Park et al., 2015; Qin et al., 2020; Yang et al., 2014b; Zhou et al., 2014b). It might also contribute to postnatal skeletal health as a source of SSPCs.

Hypertrophic chondrocytes are found at other sites, such as the cartilage to annulus fibrosus interface of the intervertebral discs and the enthesis of the tendon to bone insertion sites. These cells are not transient, and their role has not been well studied. Given the plasticity of hypertrophic chondrocytes as reviewed, it is tempting to speculate they could also have a role as progenitor cells contributing to the maintenance of the adjacent tissues, in addition to providing a junctional tissue or barrier between soft and hard tissues. However, hypertrophic chondrocytes also contribute to cartilage pathology, for example, in osteoarthritis. Whether hypertrophic chondrocytes at the different sites in healthy and pathological states are the same is unclear. This would require a thorough assessment of human tissues and animal models, perhaps leveraging on the state-of-the-art technology in single cell and spatial transcriptome to compare *Col10a1*/*COL10A1* expressing cell populations. This could also provide insight into the possible presence of hypertrophic chondrocytes in a “progenitor” state or non *Col10a1*-expressing hypertrophic chondrocytes in OA conditions, or whether in vitro differentiation protocol can progress to bona fide hypertrophic chondrocytes. Therefore, if we can understand the environment in which chondrocyte hypertrophy is prevented in healthy tissue, with the possibility of preventing or delaying ossification, or even reverting the differentiation process through the control of cellular stress signals, more innovative therapeutic options using small molecules can be achieved.

## CRediT authorship contribution statement

**Ning Chen:** Conceptualization, Writing – original draft, Writing – review & editing. **Robin W.H. Wu:** Conceptualization, Writing – original draft, Writing – review & editing. **Yan Lam:** Writing – review & editing. **Wilson C.W. Chan:** Funding acquisition, Visualization, Writing – review & editing. **Danny Chan:** Conceptualization, Funding acquisition, Visualization, Writing – original draft, Writing – review & editing.

## Declaration of competing interest

The authors declare that they have no known competing financial interests or personal relationships that could have appeared to influence the work reported in this paper.

## Data Availability

No data was used for the research described in the article.

## References

[bb0005] Aghajanian P., Xing W., Cheng S., Mohan S. (2017). Epiphyseal bone formation occurs via thyroid hormone regulation of chondrocyte to osteoblast transdifferentiation. Sci. Rep..

[bb0010] Ain N.u., Makitie O., Naz S. (2018). Autosomal recessive chondrodysplasia with severe short stature caused by a biallelic <em>COL10A1</em> variant. J. Med. Genet..

[bb0015] Akisaka T., Gay C.V. (1985). The plasma membrane and matrix vesicles of mouse growth plate chondrocytes during differentiation as revealed in freeze-fracture replicas. Am. J. Anat..

[bb0020] Akiyama H., Chaboissier M.C., Martin J.F., Schedl A., de Crombrugghe B. (2002). The transcription factor Sox9 has essential roles in successive steps of the chondrocyte differentiation pathway and is required for expression of Sox5 and Sox6. Genes Dev..

[bb0025] Ambrosi T.H., Scialdone A., Graja A., Gohlke S., Jank A.M., Bocian C., Woelk L., Fan H., Logan D.W., Schürmann A., Saraiva L.R., Schulz T.J. (2017). Adipocyte accumulation in the bone marrow during obesity and aging impairs stem cell-based hematopoietic and bone regeneration. Cell Stem Cell.

[bb0030] Anderson H.C. (1969). Vesicles associated with calcification in the matrix of epiphyseal cartilage. J. Cell Biol..

[bb0035] Anderson C.E., Parker J. (1966). Invasion and resorption in enchondral ossification. An electron microscopic study. J. Bone Joint Surg. Am..

[bb0040] Arnold M.A., Kim Y., Czubryt M.P., Phan D., McAnally J., Qi X., Shelton J.M., Richardson J.A., Bassel-Duby R., Olson E.N. (2007). MEF2C transcription factor controls chondrocyte hypertrophy and bone development. Dev. Cell.

[bb0045] Arra M., Swarnkar G., Ke K., Otero J.E., Ying J., Duan X., Maruyama T., Rai M.F., O’Keefe R.J., Mbalaviele G., Shen J., Abu-Amer Y. (2020). LDHA-mediated ROS generation in chondrocytes is a potential therapeutic target for osteoarthritis. Nat. Commun..

[bb0050] Bateman J.F., Freddi S., Nattrass G., Savarirayan R. (2003). Tissue-specific RNA surveillance? Nonsense-mediated mRNA decay causes collagen X haploinsufficiency in Schmid metaphyseal chondrodysplasia cartilage. Hum. Mol. Genet..

[bb0055] Bateman J.F., Wilson R., Freddi S., Lamandé S.R., Savarirayan R. (2005). Mutations of COL10A1 in Schmid metaphyseal chondrodysplasia. Hum. Mutat..

[bb0060] Bateman J.F., Boot-Handford R.P., Lamandé S.R. (2009). Genetic diseases of connective tissues: cellular and extracellular effects of ECM mutations. Nat. Rev. Genet..

[bb0065] Bendall A.J., Hu G., Levi G., Abate-Shen C. (2003). Dlx5 regulates chondrocyte differentiation at multiple stages. Int. J. Dev. Biol..

[bb0070] Benjamin M., McGonagle D. (2009). Entheses: tendon and ligament attachment sites. Scand. J. Med. Sci. Sports.

[bb0075] Benjamin M., Kumai T., Milz S., Boszczyk B.M., Boszczyk A.A., Ralphs J.R. (2002). The skeletal attachment of tendons–tendon “entheses”. Comp. Biochem. Physiol. A Mol. Integr. Physiol..

[bb0080] Benninghoff A. (1925). Form und Bau der Gelenkknorpel in ihren Beziehungen zur Funktion. Z. Zellforsch. Mikrosk. Anat..

[bb0085] Bentley G., Greer R.B. (1970). The fate of chondrocytes in endochondral ossification in the rabbit. J. Bone Joint Surg. Br..

[bb0090] Bentovim L., Amarilio R., Zelzer E. (2012). HIF1α is a central regulator of collagen hydroxylation and secretion under hypoxia during bone development. Development.

[bb0095] Benya P.D., Shaffer J.D. (1982). Dedifferentiated chondrocytes reexpress the differentiated collagen phenotype when cultured in agarose gels. Cell.

[bb0100] Benya P.D., Padilla S.R., Nimni M.E. (1978). Independent regulation of collagen types by chondrocytes during the loss of differentiated function in culture. Cell.

[bb0105] Benya P.D., Brown P.D., Padilla S.R. (1988). Microfilament modification by dihydrocytochalasin B causes retinoic acid-modulated chondrocytes to reexpress the differentiated collagen phenotype without a change in shape. J. Cell Biol..

[bb0110] Berg R.A., Prockop D.J. (1973). The thermal transition of a non-hydroxylated form of collagen. Evidence for a role for hydroxyproline in stabilizing the triple-helix of collagen. Biochem. Biophys. Res. Commun..

[bb0115] Blair H.C., Teitelbaum S.L., Ghiselli R., Gluck S. (1989). Osteoclastic bone resorption by a polarized vacuolar proton pump. Science.

[bb0120] Blair H.C., Zaidi M., Schlesinger P.H. (2002). Mechanisms balancing skeletal matrix synthesis and degradation. Biochem. J..

[bb0125] Böhm A.M., Dirckx N., Tower R.J., Peredo N., Vanuytven S., Theunis K., Nefyodova E., Cardoen R., Lindner V., Voet T., Van Hul M., Maes C. (2019). Activation of skeletal stem and progenitor cells for bone regeneration is driven by PDGFRβ signaling. Dev. Cell.

[bb0130] Boneski P.K., Madhu V., Tomlinson R.E., Shapiro I.M., van de Wetering K., Risbud M.V. (2022). Abcc6 null mice-a model for mineralization disorder PXE shows vertebral osteopenia without enhanced intervertebral disc calcification with aging. Front. Cell Dev. Biol..

[bb0135] Boot-Handford R.P., Briggs M.D. (2010). The unfolded protein response and its relevance to connective tissue diseases. Cell Tissue Res..

[bb0140] Bord S., Horner A., Hembry R.M., Compston J.E. (1998). Stromelysin-1 (MMP-3) and stromelysin-2 (MMP-10) expression in developing human bone: potential roles in skeletal development. Bone.

[bb0145] Boyan B.D., Schwartz Z., Swain L.D., Carnes D.L., Zislis T. (1988). Differential expression of phenotype by resting zone and growth region costochondral chondrocytes in vitro. Bone.

[bb0150] Boyde A., Jones S.J. (1983). Back-scattered electron imaging of skeletal tissues. Metab. Bone Dis. Relat. Res..

[bb0155] Bradley E.W., Carpio L.R., Westendorf J.J. (2013). Histone deacetylase 3 suppression increases PH domain and leucine-rich repeat phosphatase (Phlpp)1 expression in chondrocytes to suppress Akt signaling and matrix secretion. J. Biol. Chem..

[bb0160] Broom N.D., Myers D.B. (1980). A study of the structural response of wet hyaline cartilage to various loading situations. Connect. Tissue Res..

[bb0165] Buckwalter J.A., Mower D., Ungar R., Schaeffer J., Ginsberg B. (1986). Morphometric analysis of chondrocyte hypertrophy. J. Bone Joint Surg. Am..

[bb0170] Buckwalter J.A., Mower D., Schaeffer J. (1987). Differences in matrix vesicle concentration among growth plate zones. J. Orthop. Res..

[bb0175] Cecil R.N.A., Clarke Anderson H. (1978). Freeze-fracture studies of matrix vesicle calcification in epiphyseal growth plate. Metab. Bone Dis. Relat. Res..

[bb0180] Chan D., Jacenko O. (1998). Phenotypic and biochemical consequences of collagen X mutations in mice and humans. Matrix Biol..

[bb0185] Chan D., Cole W.G., Rogers J.G., Bateman J.F. (1995). Type X collagen multimer assembly in vitro is prevented by a Gly618 to Val mutation in the alpha 1(X) NC1 domain resulting in Schmid metaphyseal chondrodysplasia. J. Biol. Chem..

[bb0190] Chan D., Weng Y.M., Hocking A.M., Golub S., McQuillan D.J., Bateman J.F. (1996). Site-directed mutagenesis of human type X collagen: expression Of α1(X) NC1, NC2, and helical mutations in vitro and in transfected cells*. J. Biol. Chem..

[bb0195] Chan D., Weng Y.M., Graham H.K., Sillence D.O., Bateman J.F. (1998). A nonsense mutation in the carboxyl-terminal domain of type X collagen causes haploinsufficiency in schmid metaphyseal chondrodysplasia. J. Clin. Invest..

[bb0200] Chan D., Ho M.S., Cheah K.S. (2001). Aberrant signal peptide cleavage of collagen X in Schmid metaphyseal chondrodysplasia. Implications for the molecular basis of the disease. J. Biol. Chem..

[bb0205] Chan C.K., Seo E.Y., Chen J.Y., Lo D., McArdle A., Sinha R., Tevlin R., Seita J., Vincent-Tompkins J., Wearda T., Lu W.J., Senarath-Yapa K., Chung M.T., Marecic O., Tran M., Yan K.S., Upton R., Walmsley G.G., Lee A.S., Sahoo D., Kuo C.J., Weissman I.L., Longaker M.T. (2015). Identification and specification of the mouse skeletal stem cell. Cell.

[bb0210] Chau M., Dou Z., Baroncelli M., Landman E.B., Bendre A., Kanekiyo M., Gkourogianni A., Barnes K., Ottosson L., Nilsson O. (2022). The synovial microenvironment suppresses chondrocyte hypertrophy and promotes articular chondrocyte differentiation. npj Regen. Med..

[bb0215] Chen S., Tao J., Bae Y., Jiang M.-M., Bertin T., Chen Y., Yang T., Lee B. (2013). Notch gain of function inhibits chondrocyte differentiation via Rbpj-dependent suppression of Sox9. J. Bone Miner. Res..

[bb0220] Chen Y., Wu J., Zhang S., Gao W., Liao Z., Zhou T., Li Y., Su D., Liu H., Yang X., Su P., Xu C. (2022). Hnrnpk maintains chondrocytes survival and function during growth plate development via regulating Hif1α-glycolysis axis. Cell Death Dis..

[bb0225] Cheng S., Xing W., Pourteymoor S., Schulte J., Mohan S. (2016). Conditional deletion of prolyl hydroxylase domain-containing protein 2 (Phd2) gene reveals its essential role in chondrocyte function and endochondral bone formation. Endocrinology.

[bb0230] Chin H.-J., Fisher M.C., Li Y., Ferrari D., Wang C.-K.L., Lichtler A.C., Dealy C.N., Kosher R.A. (2007). Studies on the role of Dlx5 in regulation of chondrocyte differentiation during endochondral ossification in the developing mouse limb. Develop. Growth Differ..

[bb0235] Clarkin C., Olsen B.R. (2010). On bone-forming cells and blood vessels in bone development. Cell Metab..

[bb0240] Cobb J., Dierich A., Huss-Garcia Y., Duboule D. (2006). A mouse model for human short-stature syndromes identifies <i>Shox2</i> as an upstream regulator of <i>Runx2</i> during long-bone development. Proc. Natl. Acad. Sci..

[bb0245] Coghlan R.F., Oberdorf J.A., Sienko S., Aiona M.D., Boston B.A., Connelly K.J., Bahney C., LaRouche J., Almubarak S.M., Coleman D.T., Girkontaite I., von der Mark K., Lunstrum G.P., Horton W.A. (2017). A degradation fragment of type X collagen is a real-time marker for bone growth velocity. Sci. Transl. Med..

[bb0250] Cooper K.L., Oh S., Sung Y., Dasari R.R., Kirschner M.W., Tabin C.J. (2013). Multiple phases of chondrocyte enlargement underlie differences in skeletal proportions. Nature.

[bb0255] Correa D., Hesse E., Seriwatanachai D., Kiviranta R., Saito H., Yamana K., Neff L., Atfi A., Coillard L., Sitara D., Maeda Y., Warming S., Jenkins N.A., Copeland N.G., Horne W.C., Lanske B., Baron R. (2010). Zfp521 is a target gene and key effector of parathyroid hormone-related peptide signaling in growth plate chondrocytes. Dev. Cell.

[bb0260] Costa-Mattioli M., Walter P. (2020). The integrated stress response: from mechanism to disease. Science.

[bb0265] Crelin E.S., Koch W.E. (1967). An autoradiographic study of chondrocyte transformation into chondroclasts and osteocytes during bone formation in vitro. Anat. Rec..

[bb0270] Cui L., Houston D.A., Farquharson C., MacRae V.E. (2016). Characterisation of matrix vesicles in skeletal and soft tissue mineralisation. Bone.

[bb0275] Dan H., Simsa-Maziel S., Reich A., Sela-Donenfeld D., Monsonego-Ornan E. (2012). The role of matrix gla protein in ossification and recovery of the avian growth plate. Front. Endocrinol. (Lausanne).

[bb0280] De Luca F., Barnes K.M., Uyeda J.A., De-Levi S., Abad V., Palese T., Mericq V., Baron J. (2001). Regulation of growth plate chondrogenesis by bone morphogenetic protein-2. Endocrinology.

[bb0285] Ding M., Lu Y., Abbassi S., Li F., Li X., Song Y., Geoffroy V., Im H.-J., Zheng Q. (2012). Targeting Runx2 expression in hypertrophic chondrocytes impairs endochondral ossification during early skeletal development. J. Cell. Physiol..

[bb0290] Drissi M.H., Li X., Sheu T.J., Zuscik M.J., Schwarz E.M., Puzas J.E., Rosier R.N., O’Keefe R.J. (2003). Runx2/Cbfa1 stimulation by retinoic acid is potentiated by BMP2 signaling through interaction with Smad1 on the collagen X promoter in chondrocytes. J. Cell. Biochem..

[bb0295] Dy P., Wang W., Bhattaram P., Wang Q., Wang L., Ballock R.T., Lefebvre V. (2012). Sox9 directs hypertrophic maturation and blocks osteoblast differentiation of growth plate chondrocytes. Dev. Cell.

[bb0300] Eerola I., Salminen H., Lammi P., Lammi M., von der Mark K., Vuorio E., Säämänen A.M. (1998). Type X collagen, a natural component of mouse articular cartilage: association with growth, aging, and osteoarthritis. Arthritis Rheum..

[bb0305] Enomoto H., Enomoto-Iwamoto M., Iwamoto M., Nomura S., Himeno M., Kitamura Y., Kishimoto T., Komori T. (2000). Cbfa1 is a positive regulatory factor in chondrocyte maturation. J. Biol. Chem..

[bb0310] Eyre D. (2001). Articular cartilage and changes in arthritis: collagen of articular cartilage. Arthritis Res. Ther..

[bb0315] Farnum C.E., Wilsman N.J. (1987). Morphologic stages of the terminal hypertrophic chondrocyte of growth plate cartilage. Anat. Rec..

[bb0320] Farnum C.E., Wilsman N.J. (1989). Cellular turnover at the chondro-osseous junction of growth plate cartilage: analysis by serial sections at the light microscopical level. J. Orthop. Res..

[bb0325] Farnum C.E., Wilsman N.J., Hilley H.D. (1984). An ultrastructural analysis of osteochondritic growth plate cartilage in growing swine. Vet. Pathol..

[bb0330] Feng C., Chan W.C.W., Lam Y., Wang X., Chen P., Niu B., Ng V.C.W., Yeo J.C., Stricker S., Cheah K.S.E., Koch M., Mundlos S., Ng H.H., Chan D. (2019). Lgr5 and Col22a1 Mark progenitor cells in the lineage toward juvenile articular chondrocytes. Stem Cell Rep..

[bb0335] Feng T., Zhao X., Gu P., Yang W., Wang C., Guo Q., Long Q., Liu Q., Cheng Y., Li J., Cheung C.K.Y., Wu D., Kong X., Xu Y., Ye D., Hua S., Loomes K., Xu A., Hui X. (2022). Adipocyte-derived lactate is a signalling metabolite that potentiates adipose macrophage inflammation via targeting PHD2. Nat. Commun..

[bb0340] Fernández-Iglesias Á., Fuente R., Gil-Peña H., Alonso-Durán L., Santos F., López J.M. (2021). The formation of the epiphyseal bone plate occurs via combined endochondral and intramembranous-like ossification. Int. J. Mol. Sci..

[bb0345] Ferrari D., Kosher R.A. (2002). Dlx5 is a positive regulator of chondrocyte differentiation during endochondral ossification. Dev. Biol..

[bb0350] Forouhan M., Sonntag S., Boot-Handford R.P. (2018). Carbamazepine reduces disease severity in a mouse model of metaphyseal chondrodysplasia type Schmid caused by a premature stop codon (Y632X) in the Col10a1 gene. Hum. Mol. Genet..

[bb0355] Foster J.W., Dominguez-Steglich M.A., Guioli S., Kwok C., Weller P.A., Stevanović M., Weissenbach J., Mansour S., Young I.D., Goodfellow P.N., Brook J.D., Schafer A.J. (1994). Campomelic dysplasia and autosomal sex reversal caused by mutations in an SRY-related gene. Nature.

[bb0360] Fujioka H., Wang G.J., Mizuno K., Balian G., Hurwitz S.R. (1997). Changes in the expression of type-X collagen in the fibrocartilage of rat Achilles tendon attachment during development. J. Orthop. Res..

[bb0365] Fukuta S., Oyama M., Kavalkovich K., Fu F.H., Niyibizi C. (1998). Identification of types II, IX and X collagens at the insertion site of the bovine achilles tendon. Matrix Biol..

[bb0370] Galotto M., Campanile G., Robino G., Cancedda F.D., Bianco P., Cancedda R. (1994). Hypertrophic chondrocytes undergo further differentiation to osteoblast-like cells and participate in the initial bone formation in developing chick embryo. J. Bone Miner. Res..

[bb0375] Garcia M.A., Nelson W.J., Chavez N. (2018). Cell-cell junctions organize structural and signaling networks. Cold Spring Harb. Perspect. Biol..

[bb0380] Gauci S.J., Golub S.B., Tatarczuch L., Lee E., Chan D., Walsh N.C., Little C.B., Stanton H., Lokmic Z., Sims N.A., Mackie E.J., Fosang A.J. (2019). Disrupted type II collagenolysis impairs angiogenesis, delays endochondral ossification and initiates aberrant ossification in mouse limbs. Matrix Biol..

[bb0385] Gebhard S., Hattori T., Bauer E., Schlund B., Bösl M.R., de Crombrugghe B., von der Mark K. (2008). Specific expression of Cre recombinase in hypertrophic cartilage under the control of a BAC-Col10a1 promoter. Matrix Biol..

[bb0390] Gerber H.P., Vu T.H., Ryan A.M., Kowalski J., Werb Z., Ferrara N. (1999). VEGF couples hypertrophic cartilage remodeling, ossification and angiogenesis during endochondral bone formation. Nat. Med..

[bb0395] Giovannini S., Diaz-Romero J., Aigner T., Mainil-Varlet P., Nesic D. (2010). Population doublings and percentage of S100-positive cells as predictors of in vitro chondrogenicity of expanded human articular chondrocytes. J. Cell. Physiol..

[bb0400] Giovannone D., Paul S., Schindler S., Arata C., Farmer D.J.T., Patel P., Smeeton J., Crump J.G. (2019). Programmed conversion of hypertrophic chondrocytes into osteoblasts and marrow adipocytes within zebrafish bones. eLife.

[bb0405] Giustina A., Mazziotti G., Canalis E. (2008). Growth hormone, insulin-like growth factors, and the skeleton. Endocr. Rev..

[bb0410] Grant W.T., Wang G.J., Balian G. (1987). Type X collagen synthesis during endochondral ossification in fracture repair. J. Biol. Chem..

[bb0415] Greijer A.E., van der Groep P., Kemming D., Shvarts A., Semenza G.L., Meijer G.A., van de Wiel M.A., Belien J.A., van Diest P.J., van der Wall E. (2005). Up-regulation of gene expression by hypoxia is mediated predominantly by hypoxia-inducible factor 1 (HIF-1). J. Pathol..

[bb0420] Grogan S.P., Olee T., Hiraoka K., Lotz M.K. (2008). Repression of chondrogenesis through binding of notch signaling proteins HES-1 and HEY-1 to N-box domains in the COL2A1 enhancer site. Arthritis Rheumatism.

[bb0425] Gu J., Lu Y., Li F., Qiao L., Wang Q., Li N., Borgia J.A., Deng Y., Lei G., Zheng Q. (2014). Identification and characterization of the novel Col10a1 regulatory mechanism during chondrocyte hypertrophic differentiation. Cell Death Dis..

[bb0430] Guo X., Mak K.K., Taketo M.M., Yang Y. (2009). The Wnt/beta-catenin pathway interacts differentially with PTHrP signaling to control chondrocyte hypertrophy and final maturation. PLoS One.

[bb0435] Haimov H., Shimoni E., Brumfeld V., Shemesh M., Varsano N., Addadi L., Weiner S. (2020). Mineralization pathways in the active murine epiphyseal growth plate. Bone.

[bb0440] Hallett S.A., Ono W., Ono N. (2021). The hypertrophic chondrocyte: to be or not to be. Histol. Histopathol..

[bb0445] Hanaoka H. (1976). The fate of hypertrophic chondrocytes of the epiphyseal plate. An electron microscopic study. J. Bone Joint Surg. Am..

[bb0450] Harmey D., Hessle L., Narisawa S., Johnson K.A., Terkeltaub R., Millán J.L. (2004). Concerted regulation of inorganic pyrophosphate and osteopontin by akp2, enpp1, and ank: an integrated model of the pathogenesis of mineralization disorders. Am. J. Pathol..

[bb0455] Haseeb A., Kc R., Angelozzi M., de Charleroy C., Rux D., Tower R.J., Yao L., Pellegrino da Silva R., Pacifici M., Qin L., Lefebvre V. (2021). SOX9 keeps growth plates and articular cartilage healthy by inhibiting chondrocyte dedifferentiation/osteoblastic redifferentiation. Proc. Natl. Acad. Sci..

[bb0460] Henry S.P., Jang C.W., Deng J.M., Zhang Z., Behringer R.R., de Crombrugghe B. (2009). Generation of aggrecan-CreERT2 knockin mice for inducible Cre activity in adult cartilage. Genesis.

[bb0465] Hessle L., Johnson K.A., Anderson H.C., Narisawa S., Sali A., Goding J.W., Terkeltaub R., Millán J.L. (2002). Tissue-nonspecific alkaline phosphatase and plasma cell membrane glycoprotein-1 are central antagonistic regulators of bone mineralization. Proc. Natl. Acad. Sci..

[bb0470] Hetz C., Zhang K., Kaufman R.J. (2020). Mechanisms, regulation and functions of the unfolded protein response. Nat. Rev. Mol. Cell Biol..

[bb0475] Hirata M., Kugimiya F., Fukai A., Ohba S., Kawamura N., Ogasawara T., Kawasaki Y., Saito T., Yano F., Ikeda T., Nakamura K., Chung U.I., Kawaguchi H. (2009). C/EBPbeta promotes transition from proliferation to hypertrophic differentiation of chondrocytes through transactivation of p57. PLoS One.

[bb0480] Ho A.M., Johnson M.D., Kingsley D.M. (2000). Role of the mouse <i>ank</i> gene in control of tissue calcification and arthritis. Science.

[bb0485] Ho M.S.P., Tsang K.Y., Lo R.L.K., Susic M., Mäkitie O., Chan T.W.Y., Ng V.C.W., Sillence D.O., Boot-Handford R.P., Gibson G., Cheung K.M.C., Cole W.G., Cheah K.S.E., Chan D. (2007). COL10A1 nonsense and frame-shift mutations have a gain-of-function effect on the growth plate in human and mouse metaphyseal chondrodysplasia type Schmid. Hum. Mol. Genet..

[bb0490] Hosaka Y., Saito T., Sugita S., Hikata T., Kobayashi H., Fukai A., Taniguchi Y., Hirata M., Akiyama H., Chung U.-i., Kawaguchi H. (2013). Notch signaling in chondrocytes modulates endochondral ossification and osteoarthritis development. Proc. Natl. Acad. Sci..

[bb0495] Hu H., Hilton M.J., Tu X., Yu K., Ornitz D.M., Long F. (2005). Sequential roles of hedgehog and Wnt signaling in osteoblast development. Development.

[bb0500] Hu D.P., Ferro F., Yang F., Taylor A.J., Chang W., Miclau T., Marcucio R.S., Bahney C.S. (2017). Cartilage to bone transformation during fracture healing is coordinated by the invading vasculature and induction of the core pluripotency genes. Development.

[bb0505] Hunziker E.B. (1994). Mechanism of longitudinal bone growth and its regulation by growth plate chondrocytes. Microsc. Res. Tech..

[bb0510] Hunziker E.B., Schenk R.K. (1989). Physiological mechanisms adopted by chondrocytes in regulating longitudinal bone growth in rats. J. Physiol..

[bb0515] Hunziker E.B., Herrmann W., Schenk R.K., Mueller M., Moor H. (1984). Cartilage ultrastructure after high pressure freezing, freeze substitution, and low temperature embedding. I. Chondrocyte ultrastructure–implications for the theories of mineralization and vascular invasion. J. Cell Biol..

[bb0520] Hunziker E.B., Schenk R.K., Cruz-Orive L.M. (1987). Quantitation of chondrocyte performance in growth-plate cartilage during longitudinal bone growth. J. Bone Joint Surg. Am..

[bb0525] Ikeda T., Nomura S., Yamaguchi A., Suda T., Yoshiki S. (1992). In situ hybridization of bone matrix proteins in undecalcified adult rat bone sections. J. Histochem. Cytochem..

[bb0530] Ikegawa S., Nishimura G., Nagai T., Hasegawa T., Ohashi H., Nakamura Y. (1998). Mutation of the type X collagen gene (COL10A1) causes spondylometaphyseal dysplasia. Am. J. Hum. Genet..

[bb0535] Inada M., Yasui T., Nomura S., Miyake S., Deguchi K., Himeno M., Sato M., Yamagiwa H., Kimura T., Yasui N., Ochi T., Endo N., Kitamura Y., Kishimoto T., Komori T. (1999). Maturational disturbance of chondrocytes in Cbfa1-deficient mice. Dev. Dyn..

[bb0540] Inada M., Wang Y., Byrne M.H., Rahman M.U., Miyaura C., López-Otín C., Krane S.M. (2004). Critical roles for collagenase-3 (Mmp13) in development of growth plate cartilage and in endochondral ossification. Proc. Natl. Acad. Sci..

[bb0545] Ionescu A., Kozhemyakina E., Nicolae C., Kaestner K.H., Olsen B.R., Lassar A.B. (2012). FoxA family members are crucial regulators of the hypertrophic chondrocyte differentiation program. Dev. Cell.

[bb0550] Jacenko O., LuValle P.A., Olsen B.R. (1993). Spondylometaphyseal dysplasia in mice carrying a dominant negative mutation in a matrix protein specific for cartilage-to-bone transition. Nature.

[bb0555] Javaheri B., Caetano-Silva S.P., Kanakis I., Bou-Gharios G., Pitsillides A.A. (2018). The Chondro-osseous continuum: is it possible to unlock the potential assigned within?. Front. Bioeng. Biotechnol..

[bb0560] Jiang J., Hui C.C. (2008). Hedgehog signaling in development and cancer. Dev. Cell.

[bb0565] Jing Y., Jing J., Wang K., Chan K., Harris S.E., Hinton R.J., Feng J.Q. (2018). Vital roles of β-catenin in trans-differentiation of chondrocytes to bone cells. Int. J. Biol. Sci..

[bb0570] Johnson K.A., Hessle L., Vaingankar S., Wennberg C., Mauro S., Narisawa S., Goding J.W., Sano K., Millan J.L., Terkeltaub R. (2000). Osteoblast tissue-nonspecific alkaline phosphatase antagonizes and regulates PC-1. Am. J. Phys. Regul. Integr. Comp. Phys..

[bb0575] Kahn A.J., Simmons D.J. (1977). Chondrocyte-to-osteocyte transformation in grafts of perichondrium-free epiphyseal cartilage. Clin. Orthop. Relat. Res..

[bb0580] Kang S.W., Yoo S.P., Kim B.S. (2007). Effect of chondrocyte passage number on histological aspects of tissue-engineered cartilage. Biomed. Mater. Eng..

[bb0585] Karaplis A.C., Luz A., Glowacki J., Bronson R.T., Tybulewicz V.L., Kronenberg H.M., Mulligan R.C. (1994). Lethal skeletal dysplasia from targeted disruption of the parathyroid hormone-related peptide gene. Genes Dev..

[bb0590] Kerschnitzki M., Akiva A., Shoham A.B., Koifman N., Shimoni E., Rechav K., Arraf A.A., Schultheiss T.M., Talmon Y., Zelzer E., Weiner S., Addadi L. (2016). Transport of membrane-bound mineral particles in blood vessels during chicken embryonic bone development. Bone.

[bb0595] Killian M.L. (2022). Growth and mechanobiology of the tendon-bone enthesis. Semin. Cell Dev. Biol..

[bb0600] Kim I.S., Otto F., Zabel B., Mundlos S. (1999). Regulation of chondrocyte differentiation by Cbfa1. Mech. Dev..

[bb0605] Kirsch T., Nah H.D., Shapiro I.M., Pacifici M. (1997). Regulated production of mineralization-competent matrix vesicles in hypertrophic chondrocytes. J. Cell Biol..

[bb0610] Kishimoto K., Kitazawa R., Kurosaka M., Maeda S., Kitazawa S. (2006). Expression profile of genes related to osteoclastogenesis in mouse growth plate and articular cartilage. Histochem. Cell Biol..

[bb0615] Kobayashi T., Chung U.I., Schipani E., Starbuck M., Karsenty G., Katagiri T., Goad D.L., Lanske B., Kronenberg H.M. (2002). PTHrP and Indian hedgehog control differentiation of growth plate chondrocytes at multiple steps. Development.

[bb0620] Kodama J., Wilkinson K.J., Iwamoto M., Otsuru S., Enomoto-Iwamoto M. (2022). The role of hypertrophic chondrocytes in regulation of the cartilage-to-bone transition in fracture healing. Bone Rep.

[bb0625] Kohn A., Rutkowski T.P., Liu Z., Mirando A.J., Zuscik M.J., O’Keefe R.J., Hilton M.J. (2015). Notch signaling controls chondrocyte hypertrophy via indirect regulation of Sox9. Bone Res..

[bb0630] Komori T. (2022). Whole aspect of Runx2 functions in skeletal development. Int. J. Mol. Sci..

[bb0635] Komori T., Yagi H., Nomura S., Yamaguchi A., Sasaki K., Deguchi K., Shimizu Y., Bronson R.T., Gao Y.H., Inada M., Sato M., Okamoto R., Kitamura Y., Yoshiki S., Kishimoto T. (1997). Targeted disruption of Cbfa1 results in a complete lack of bone formation owing to maturational arrest of osteoblasts. Cell.

[bb0640] Kong R.Y., Kwan K.M., Lau E.T., Thomas J.T., Boot-Handford R.P., Grant M.E., Cheah K.S. (1993). Intron-exon structure, alternative use of promoter and expression of the mouse collagen X gene, Col10a-1. Eur. J. Biochem..

[bb0645] Kozhemyakina E., Cohen T., Yao T.P., Lassar A.B. (2009). Parathyroid hormone-related peptide represses chondrocyte hypertrophy through a protein phosphatase 2A/histone deacetylase 4/MEF2 pathway. Mol. Cell. Biol..

[bb0650] Kozhemyakina E., Lassar A.B., Zelzer E. (2015). A pathway to bone: signaling molecules and transcription factors involved in chondrocyte development and maturation. Development.

[bb0655] Kozhemyakina E., Zhang M., Ionescu A., Ayturk U.M., Ono N., Kobayashi A., Kronenberg H., Warman M.L., Lassar A.B. (2015). Identification of a Prg4-expressing articular cartilage progenitor cell population in mice. Arthritis Rheumatol..

[bb0660] Kronenberg H.M. (2003). Developmental regulation of the growth plate. Nature.

[bb0665] Kung L.H., Rajpar M.H., Briggs M.D., Boot-Handford R.P. (2012). Hypertrophic chondrocytes have a limited capacity to cope with increases in endoplasmic reticulum stress without triggering the unfolded protein response. J. Histochem. Cytochem..

[bb0670] Kurosaki T., Popp M.W., Maquat L.E. (2019). Quality and quantity control of gene expression by nonsense-mediated mRNA decay. Nat. Rev. Mol. Cell Biol..

[bb0675] Kwan K.M., Pang M.K., Zhou S., Cowan S.K., Kong R.Y., Pfordte T., Olsen B.R., Sillence D.O., Tam P.P., Cheah K.S. (1997). Abnormal compartmentalization of cartilage matrix components in mice lacking collagen X: implications for function. J. Cell Biol..

[bb0680] Lazarus S., Tseng H.W., Lawrence F., Woodruff M.A., Duncan E.L., Pettit A.R. (2017). Characterization of normal murine carpal bone development prompts re-evaluation of pathologic osteolysis as the cause of human carpal-tarsal osteolysis disorders. Am. J. Pathol..

[bb0685] Lee E.R., Murphy G., El-Alfy M., Davoli M.A., Lamplugh L., Docherty A.J., Leblond C.P. (1999). Active gelatinase B is identified by histozymography in the cartilage resorption sites of developing long bones. Dev. Dyn..

[bb0690] Lee M.H., Kim Y.J., Kim H.J., Park H.D., Kang A.R., Kyung H.M., Sung J.H., Wozney J.M., Kim H.J., Ryoo H.M. (2003). BMP-2-induced Runx2 expression is mediated by Dlx5, and TGF-beta 1 opposes the BMP-2-induced osteoblast differentiation by suppression of Dlx5 expression. J. Biol. Chem..

[bb0695] Lee M.-H., Kim Y.-J., Yoon W.-J., Kim J.-I., Kim B.-G., Hwang Y.-S., Wozney J.M., Chi X.-Z., Bae S.-C., Choi K.-Y., Cho J.-Y., Choi J.-Y., Ryoo H.-M. (2005). Dlx5 specifically regulates Runx2 type II expression by binding to homeodomain-response elements in the Runx2 distal promoter*. J. Biol. Chem..

[bb0700] Lee J., Lee J.-Y., Chae B.-C., Jang J., Lee E., Son Y. (2017). Fully dedifferentiated chondrocytes expanded in specific mesenchymal stem cell growth medium with FGF2 obtains mesenchymal stem cell phenotype in vitro but retains chondrocyte phenotype in vivo. Cell Transplant..

[bb0705] Lefebvre V., Peeters-Joris C., Vaes G. (1990). Production of collagens, collagenase and collagenase inhibitor during the dedifferentiation of articular chondrocytes by serial subcultures. Biochim. Biophys. Acta - Mol. Cell Res..

[bb0710] Lefebvre V., Angelozzi M., Haseeb A. (2019). SOX9 in cartilage development and disease. Curr. Opin. Cell Biol..

[bb0715] Lemare F., Steimberg N., Le Griel C., Demignot S., Adolphe M. (1998). Dedifferentiated chondrocytes cultured in alginate beads: restoration of the differentiated phenotype and of the metabolic responses to interleukin-1β. J. Cell. Physiol..

[bb0720] Lengner C.J., Hassan M.Q., Serra R.W., Lepper C., van Wijnen A.J., Stein J.L., Lian J.B., Stein G.S. (2005). Nkx3.2-mediated repression of Runx2 promotes Chondrogenic differentiation*. J. Biol. Chem..

[bb0725] Lin Z., Fitzgerald J.B., Xu J., Willers C., Wood D., Grodzinsky A.J., Zheng M.H. (2008). Gene expression profiles of human chondrocytes during passaged monolayer cultivation. J. Orthop. Res..

[bb0730] Lin Z., McClure M.J., Zhao J., Ramey A.N., Asmussen N., Hyzy S.L., Schwartz Z., Boyan B.D. (2018). MicroRNA contents in matrix vesicles produced by growth plate chondrocytes are cell maturation dependent. Sci. Rep..

[bb0735] Long F., Chung U.I., Ohba S., McMahon J., Kronenberg H.M., McMahon A.P. (2004). Ihh signaling is directly required for the osteoblast lineage in the endochondral skeleton. Development.

[bb0740] Long J.T., Leinroth A., Liao Y., Ren Y., Mirando A.J., Nguyen T., Guo W., Sharma D., Rouse D., Wu C., Cheah K.S.E., Karner C.M., Hilton M.J. (2022). Hypertrophic chondrocytes serve as a reservoir for marrow-associated skeletal stem and progenitor cells, osteoblasts, and adipocytes during skeletal development. eLife.

[bb0745] Luo G., Ducy P., McKee M.D., Pinero G.J., Loyer E., Behringer R.R., Karsenty G. (1997). Spontaneous calcification of arteries and cartilage in mice lacking matrix GLA protein. Nature.

[bb0750] Lutfi A.M. (1971). The fate of chondrocytes during cartilage erosion in the growing tibia in the domestic fowl (Gallus domesticus). Acta Anat. (Basel).

[bb0755] Ma S.K.Y., Chan A.S.F., Rubab A., Chan W.C.W., Chan D. (2020). Extracellular matrix and cellular plasticity in musculoskeletal development. Front Cell Dev. Biol..

[bb0760] MacLean H.E., Guo J., Knight M.C., Zhang P., Cobrinik D., Kronenberg H.M. (2004). The cyclin-dependent kinase inhibitor p57(Kip2) mediates proliferative actions of PTHrP in chondrocytes. J. Clin. Invest..

[bb0765] Maes C., Araldi E., Haigh K., Khatri R., Van Looveren R., Giaccia A.J., Haigh J.J., Carmeliet G., Schipani E. (2012). VEGF-independent cell-autonomous functions of HIF-1α regulating oxygen consumption in fetal cartilage are critical for chondrocyte survival. J. Bone Miner. Res..

[bb0770] Majeska R.J., Wuthier R.E. (1975). Studies on matrix vesicles isolated from chick epiphyseal cartilage association of pyrophosphatase and ATPase activities with alkaline phosphatase. Biochim. Biophys. Acta - Biomembr..

[bb0775] Mak K.K., Kronenberg H.M., Chuang P.T., Mackem S., Yang Y. (2008). Indian hedgehog signals independently of PTHrP to promote chondrocyte hypertrophy. Development.

[bb0780] Meyer J.L. (1984). Can biological calcification occur in the presence of pyrophosphate?. Arch. Biochem. Biophys..

[bb0785] Miao D., Bai X., Panda D.K., Karaplis A.C., Goltzman D., McKee M.D. (2004). Cartilage abnormalities are associated with abnormal Phex expression and with altered matrix protein and MMP-9 localization in Hyp mice. Bone.

[bb0790] Minina E., Wenzel H.M., Kreschel C., Karp S., Gaffield W., McMahon A.P., Vortkamp A. (2001). BMP and Ihh/PTHrP signaling interact to coordinate chondrocyte proliferation and differentiation. Development.

[bb0795] Mizuhashi K., Ono W., Matsushita Y., Sakagami N., Takahashi A., Saunders T.L., Nagasawa T., Kronenberg H.M., Ono N. (2018). Resting zone of the growth plate houses a unique class of skeletal stem cells. Nature.

[bb0800] Morris D.C., Vaananen H.K., Anderson H.C. (1983). Matrix vesicle calcification in rat epiphyseal growth plate cartilage prepared anhydrously for electron microscopy. Metab. Bone Dis. Relat. Res..

[bb0805] Moskalewski S., Malejczyk J. (1989). Bone formation following intrarenal transplantation of isolated murine chondrocytes: chondrocyte-bone cell transdifferentiation?. Development.

[bb0810] Mullan L.A., Mularczyk E.J., Kung L.H., Forouhan M., Wragg J.M., Goodacre R., Bateman J.F., Swanton E., Briggs M.D., Boot-Handford R.P. (2017). Increased intracellular proteolysis reduces disease severity in an ER stress-associated dwarfism. J. Clin. Invest..

[bb0815] Mwale F., Tchetina E., Wu C.W., Poole A.R. (2002). The assembly and remodeling of the extracellular matrix in the growth plate in relationship to mineral deposition and cellular hypertrophy: an in situ study of collagens II and IX and proteoglycan. J. Bone Miner. Res..

[bb0820] Neuman W.F., Neuman M.W. (1953). The nature of the mineral phase of bone. Chem. Rev..

[bb0825] Newton P.T., Xie M., Medvedeva E.V., Sävendahl L., Chagin A.S. (2018). Activation of mTORC1 in chondrocytes does not affect proliferation or differentiation, but causes the resting zone of the growth plate to become disordered. Bone Rep..

[bb0830] Newton P.T., Li L., Zhou B., Schweingruber C., Hovorakova M., Xie M., Sun X., Sandhow L., Artemov A.V., Ivashkin E., Suter S., Dyachuk V., El Shahawy M., Gritli-Linde A., Bouderlique T., Petersen J., Mollbrink A., Lundeberg J., Enikolopov G., Qian H., Fried K., Kasper M., Hedlund E., Adameyko I., Sävendahl L., Chagin A.S. (2019). A radical switch in clonality reveals a stem cell niche in the epiphyseal growth plate. Nature.

[bb0835] Nilsson O., Parker E.A., Hegde A., Chau M., Barnes K.M., Baron J. (2007). Gradients in bone morphogenetic protein-related gene expression across the growth plate. J. Endocrinol..

[bb0840] Ninomiya Y., Gordon M., van der Rest M., Schmid T., Linsenmayer T., Olsen B.R. (1986). The developmentally regulated type X collagen gene contains a long open reading frame without introns. J. Biol. Chem..

[bb0845] Nishida T., Kubota S., Aoyama E., Takigawa M. (2013). Impaired glycolytic metabolism causes chondrocyte hypertrophy-like changes via promotion of phospho-Smad1/5/8 translocation into nucleus. Osteoarthr. Cartil..

[bb0850] Niyibizi C., Sagarrigo Visconti C., Gibson G., Kavalkovich K. (1996). Identification and immunolocalization of type X collagen at the ligament-bone interface. Biochem. Biophys. Res. Commun..

[bb0855] Nurminsky D., Magee C., Faverman L., Nurminskaya M. (2007). Regulation of chondrocyte differentiation by actin-severing protein adseverin. Dev. Biol..

[bb0860] Ortega N., Behonick D.J., Werb Z. (2004). Matrix remodeling during endochondral ossification. Trends Cell Biol..

[bb0865] Ortega N., Wang K., Ferrara N., Werb Z., Vu T.H. (2010). Complementary interplay between matrix metalloproteinase-9, vascular endothelial growth factor and osteoclast function drives endochondral bone formation. Dis. Model. Mech..

[bb0870] Otto F., Thornell A.P., Crompton T., Denzel A., Gilmour K.C., Rosewell I.R., Stamp G.W., Beddington R.S., Mundlos S., Olsen B.R., Selby P.B., Owen M.J. (1997). Cbfa1, a candidate gene for cleidocranial dysplasia syndrome, is essential for osteoblast differentiation and bone development. Cell.

[bb0875] Pakos-Zebrucka K., Koryga I., Mnich K., Ljujic M., Samali A., Gorman A.M. (2016). The integrated stress response. EMBO Rep..

[bb0880] Park J., Gebhardt M., Golovchenko S., Perez-Branguli F., Hattori T., Hartmann C., Zhou X., deCrombrugghe B., Stock M., Schneider H., von der Mark K. (2015). Dual pathways to endochondral osteoblasts: a novel chondrocyte-derived osteoprogenitor cell identified in hypertrophic cartilage. Biol. Open.

[bb0885] Park S., Bello A., Arai Y., Ahn J., Kim D., Cha K.Y., Baek I., Park H., Lee S.H. (2021). Functional duality of chondrocyte hypertrophy and biomedical application trends in osteoarthritis. Pharmaceutics.

[bb0890] Parreno J., Nabavi Niaki M., Andrejevic K., Jiang A., Wu P.-h., Kandel R.A. (2017). Interplay between cytoskeletal polymerization and the chondrogenic phenotype in chondrocytes passaged in monolayer culture. J. Anat..

[bb0895] Patra D., Xing X., Davies S., Bryan J., Franz C., Hunziker E.B., Sandell L.J. (2007). Site-1 protease is essential for endochondral bone formation in mice. J. Cell Biol..

[bb0900] Peng X.D., Xu P.Z., Chen M.L., Hahn-Windgassen A., Skeen J., Jacobs J., Sundararajan D., Chen W.S., Crawford S.E., Coleman K.G., Hay N. (2003). Dwarfism, impaired skin development, skeletal muscle atrophy, delayed bone development, and impeded adipogenesis in mice lacking Akt1 and Akt2. Genes Dev..

[bb0905] Pfander D., Kobayashi T., Knight M.C., Zelzer E., Chan D.A., Olsen B.R., Giaccia A.J., Johnson R.S., Haase V.H., Schipani E. (2004). Deletion of Vhlh in chondrocytes reduces cell proliferation and increases matrix deposition during growth plate development. Development.

[bb0910] Phimphilai M., Zhao Z., Boules H., Roca H., Franceschi R.T. (2006). BMP signaling is required for RUNX2-dependent induction of the osteoblast phenotype. J. Bone Miner. Res..

[bb0915] Pollesello P., de Bernard B., Grandolfo M., Paoletti S., Vittur F., Kvam B.J. (1991). Energy state of chondrocytes assessed by 31P-NMR studies of preosseous cartilage. Biochem. Biophys. Res. Commun..

[bb0920] Poole C.A. (1997). Articular cartilage chondrons: form, function and failure. J. Anat..

[bb0925] Prein C., Beier F., Olsen B.R. (2019). Current Topics in Developmental Biology.

[bb0930] Provot S., Kempf H., Murtaugh L.C., Chung U.-i., Kim D.-W., Chyung J., Kronenberg H.M., Lassar A.B. (2006). Nkx3.2/Bapx1 acts as a negative regulator of chondrocyte maturation. Development.

[bb0935] Qin X., Jiang Q., Nagano K., Moriishi T., Miyazaki T., Komori H., Ito K., Mark K.V., Sakane C., Kaneko H., Komori T. (2020). Runx2 is essential for the transdifferentiation of chondrocytes into osteoblasts. PLoS Genet..

[bb0940] Rajpar M.H., McDermott B., Kung L., Eardley R., Knowles L., Heeran M., Thornton D.J., Wilson R., Bateman J.F., Poulsom R., Arvan P., Kadler K.E., Briggs M.D., Boot-Handford R.P. (2009). Targeted induction of endoplasmic reticulum stress induces cartilage pathology. PLoS Genet..

[bb0945] Roach H.I., Erenpreisa J., Aigner T. (1995). Osteogenic differentiation of hypertrophic chondrocytes involves asymmetric cell divisions and apoptosis. J. Cell Biol..

[bb0950] Rodríguez J.P., González M., Ríos S., Cambiazo V. (2004). Cytoskeletal organization of human mesenchymal stem cells (MSC) changes during their osteogenic differentiation. J. Cell. Biochem..

[bb0955] Rokutanda S., Fujita T., Kanatani N., Yoshida C.A., Komori H., Liu W., Mizuno A., Komori T. (2009). Akt regulates skeletal development through GSK3, mTOR, and FoxOs. Dev. Biol..

[bb0960] Rooney P., Grant M.E., McClure J. (1992). Endochondral ossification and de novo collagen synthesis during repair of the rat Achilles tendon. Matrix.

[bb0965] Rosati R., Horan G.S.B., Pinero G.J., Garofalo S., Keene D.R., Horton W.A., Vuorio E., de Crombrugghe B., Behringer R.R. (1994). Normal long bone growth and development in type X collagen-null mice. Nat. Genet..

[bb0970] Rutkowski T.P., Kohn A., Sharma D., Ren Y., Mirando A.J., Hilton M.J. (2016). HES factors regulate specific aspects of chondrogenesis and chondrocyte hypertrophy during cartilage development. J. Cell Sci..

[bb0975] Saito A., Hino S.-i., Murakami T., Kanemoto S., Kondo S., Saitoh M., Nishimura R., Yoneda T., Furuichi T., Ikegawa S., Ikawa M., Okabe M., Imaizumi K. (2009). Regulation of endoplasmic reticulum stress response by a BBF2H7-mediated Sec23a pathway is essential for chondrogenesis. Nat. Cell Biol..

[bb0980] Sakamoto A., Chen M., Kobayashi T., Kronenberg H.M., Weinstein L.S. (2005). Chondrocyte-specific knockout of the G protein G(s)alpha leads to epiphyseal and growth plate abnormalities and ectopic chondrocyte formation. J. Bone Miner. Res..

[bb0985] Sasagawa S., Takemori H., Uebi T., Ikegami D., Hiramatsu K., Ikegawa S., Yoshikawa H., Tsumaki N. (2012). SIK3 is essential for chondrocyte hypertrophy during skeletal development in mice. Development.

[bb0990] Sato H., Takino T., Okada Y., Cao J., Shinagawa A., Yamamoto E., Seiki M. (1994). A matrix metalloproteinase expressed on the surface of invasive tumour cells. Nature.

[bb0995] Sato S., Kimura A., Ozdemir J., Asou Y., Miyazaki M., Jinno T., Ae K., Liu X., Osaki M., Takeuchi Y., Fukumoto S., Kawaguchi H., Haro H., Shinomiya K., Karsenty G., Takeda S. (2008). The distinct role of the Runx proteins in chondrocyte differentiation and intervertebral disc degeneration: findings in murine models and in human disease. Arthritis Rheum..

[bb1000] Schejter E.D., Baylies M.K. (2010). Born to run: creating the muscle fiber. Curr. Opin. Cell Biol..

[bb1005] Schipani E., Ryan H.E., Didrickson S., Kobayashi T., Knight M., Johnson R.S. (2001). Hypoxia in cartilage: HIF-1alpha is essential for chondrocyte growth arrest and survival. Genes Dev..

[bb1010] Semenza Gregg L. (2012). Hypoxia-inducible factors in physiology and medicine. Cell.

[bb1015] Shah M., Gburcik V., Reilly P., Sankey R.A., Emery R.J., Clarkin C.E., Pitsillides A.A. (2015). Local origins impart conserved bone type-related differences in human osteoblast behaviour. Eur. Cell Mater..

[bb1020] Shang X., Wang J., Luo Z., Wang Y., Morandi M.M., Marymont J.V., Hilton M.J., Dong Y. (2016). Notch signaling indirectly promotes chondrocyte hypertrophy via regulation of BMP signaling and cell cycle arrest. Sci. Rep..

[bb1025] Shapiro I.M., Mansfield K.D., Evans S.M., Lord E.M., Koch C.J. (1997). Chondrocytes in the endochondral growth cartilage are not hypoxic. Am. J. Phys..

[bb1030] Shimomura Y., Yoneda T., Suzuki F. (1975). Osteogenesis by chondrocytes from growth cartilage of rat rib. Calcif. Tissue Res..

[bb1035] Shimoyama A., Wada M., Ikeda F., Hata K., Matsubara T., Nifuji A., Noda M., Amano K., Yamaguchi A., Nishimura R., Yoneda T. (2007). Ihh/Gli2 signaling promotes osteoblast differentiation by regulating Runx2 expression and function. Mol. Biol. Cell.

[bb1040] Stegen S., Laperre K., Eelen G., Rinaldi G., Fraisl P., Torrekens S., Van Looveren R., Loopmans S., Bultynck G., Vinckier S., Meersman F., Maxwell P.H., Rai J., Weis M., Eyre D.R., Ghesquière B., Fendt S.M., Carmeliet P., Carmeliet G. (2019). HIF-1α metabolically controls collagen synthesis and modification in chondrocytes. Nature.

[bb1045] Stewart M.C., Kadlcek R.M., Robbins P.D., MacLeod J.N., Ballock R.T. (2004). Expression and activity of the CDK inhibitor p57Kip2 in chondrocytes undergoing hypertrophic differentiation. J. Bone Miner. Res..

[bb1050] Stickens D., Behonick D.J., Ortega N., Heyer B., Hartenstein B., Yu Y., Fosang A.J., Schorpp-Kistner M., Angel P., Werb Z. (2004). Altered endochondral bone development in matrix metalloproteinase 13-deficient mice. Development.

[bb1055] St-Jacques B., Hammerschmidt M., McMahon A.P. (1999). Indian hedgehog signaling regulates proliferation and differentiation of chondrocytes and is essential for bone formation. Genes Dev..

[bb1060] Sugita S., Hosaka Y., Okada K., Mori D., Yano F., Kobayashi H., Taniguchi Y., Mori Y., Okuma T., Chang S.H., Kawata M., Taketomi S., Chikuda H., Akiyama H., Kageyama R., Chung U.-i., Tanaka S., Kawaguchi H., Ohba S., Saito T. (2015). Transcription factor Hes1 modulates osteoarthritis development in cooperation with calcium/calmodulin-dependent protein kinase 2. Proc. Natl. Acad. Sci..

[bb1065] Takeda S., Bonnamy J.P., Owen M.J., Ducy P., Karsenty G. (2001). Continuous expression of Cbfa1 in nonhypertrophic chondrocytes uncovers its ability to induce hypertrophic chondrocyte differentiation and partially rescues Cbfa1-deficient mice. Genes Dev..

[bb1070] Tan J.T., Kremer F., Freddi S., Bell K.M., Baker N.L., Lamandé S.R., Bateman J.F. (2008). Competency for nonsense-mediated reduction in collagen X mRNA is specified by the 3’ UTR and corresponds to the position of mutations in Schmid metaphyseal chondrodysplasia. Am. J. Hum. Genet..

[bb1075] Tan Z., Kong M., Wen S., Tsang K.Y., Niu B., Hartmann C., Chan D., Hui C.-C., Cheah K.S.E. (2020). IRX3 and IRX5 inhibit Adipogenic differentiation of hypertrophic chondrocytes and promote Osteogenesis. J. Bone Miner. Res..

[bb1080] Tan K., Stupack D.G., Wilkinson M.F. (2022). Nonsense-mediated RNA decay: an emerging modulator of malignancy. Nat. Rev. Cancer.

[bb1085] Taylor R.W., Mitchell G.K., Andrade J.L., Svoboda K.K. (2020). Expression of collagen types I, II, IX, and X in the mineralizing Turkey gastrocnemius tendon. Anat. Rec. (Hoboken).

[bb1090] Terkeltaub R.A. (2001). Inorganic pyrophosphate generation and disposition in pathophysiology. Am. J. Phys. Cell Phys..

[bb1095] Tidball J.G., Lin C. (1989). Structural changes at the myogenic cell surface during the formation of myotendinous junctions. Cell Tissue Res..

[bb1100] Tsang K.Y., Chan D., Cheslett D., Chan W.C., So C.L., Melhado I.G., Chan T.W., Kwan K.M., Hunziker E.B., Yamada Y., Bateman J.F., Cheung K.M., Cheah K.S. (2007). Surviving endoplasmic reticulum stress is coupled to altered chondrocyte differentiation and function. PLoS Biol..

[bb1105] Tsang K.Y., Chan D., Bateman J.F., Cheah K.S.E. (2010). In vivo cellular adaptation to ER stress: survival strategies with double-edged consequences. J. Cell Sci..

[bb1110] Tsang K.Y., Chan D., Cheah K.S.E. (2015). Fate of growth plate hypertrophic chondrocytes: death or lineage extension?. Develop. Growth Differ..

[bb1115] Ulici V., Hoenselaar K.D., Gillespie J.R., Beier F. (2008). The PI3K pathway regulates endochondral bone growth through control of hypertrophic chondrocyte differentiation. BMC Dev. Biol..

[bb1120] Ulici V., Hoenselaar K.D., Agoston H., McErlain D.D., Umoh J., Chakrabarti S., Holdsworth D.W., Beier F. (2009). The role of Akt1 in terminal stages of endochondral bone formation: angiogenesis and ossification. Bone.

[bb1125] van der Kraan P.M., van den Berg W.B. (2012). Chondrocyte hypertrophy and osteoarthritis: role in initiation and progression of cartilage degeneration?. Osteoarthr. Cartil..

[bb1130] van der Kraan P.M., Blaney Davidson E.N., Blom A., van den Berg W.B. (2009). TGF-beta signaling in chondrocyte terminal differentiation and osteoarthritis: modulation and integration of signaling pathways through receptor-Smads. Osteoarthr. Cartil..

[bb1135] van Donkelaar C.C., Huiskes R. (2007). The PTHrP-Ihh feedback loop in the embryonic growth plate allows PTHrP to control hypertrophy and Ihh to regulate proliferation. Biomech. Model. Mechanobiol..

[bb1140] Vega R.B., Matsuda K., Oh J., Barbosa A.C., Yang X., Meadows E., McAnally J., Pomajzl C., Shelton J.M., Richardson J.A., Karsenty G., Olson E.N. (2004). Histone deacetylase 4 controls chondrocyte hypertrophy during skeletogenesis. Cell.

[bb1145] Von Der Mark K., Gauss V., Von Der Mark H., MÜLler, P. (1977). Relationship between cell shape and type of collagen synthesised as chondrocytes lose their cartilage phenotype in culture. Nature.

[bb1150] Vortkamp A., Lee K., Lanske B., Segre G.V., Kronenberg H.M., Tabin C.J. (1996). Regulation of rate of cartilage differentiation by Indian hedgehog and PTH-related protein. Science.

[bb1155] Vu T.H., Shipley J.M., Bergers G., Berger J.E., Helms J.A., Hanahan D., Shapiro S.D., Senior R.M., Werb Z. (1998). MMP-9/gelatinase B is a key regulator of growth plate angiogenesis and apoptosis of hypertrophic chondrocytes. Cell.

[bb1160] Wagner T., Wirth J., Meyer J., Zabel B., Held M., Zimmer J., Pasantes J., Bricarelli F.D., Keutel J., Hustert E., Wolf U., Tommerup N., Schempp W., Scherer G. (1994). Autosomal sex reversal and campomelic dysplasia are caused by mutations in and around the SRY-related gene SOX9. Cell.

[bb1165] Wang J., Zhou J., Bondy C.A. (1999). Igf1 promotes longitudinal bone growth by insulin-like actions augmenting chondrocyte hypertrophy. FASEB J..

[bb1170] Wang L., Huang J., Moore D.C., Zuo C., Wu Q., Xie L., von der Mark K., Yuan X., Chen D., Warman M.L., Ehrlich M.G., Yang W. (2017). SHP2 regulates the osteogenic fate of growth plate hypertrophic chondrocytes. Sci. Rep..

[bb1175] Wang C., Tan Z., Niu B., Tsang K.Y., Tai A., Chan W.C.W., Lo R.L.K., Leung K.K.H., Dung N.W.F., Itoh N., Zhang M.Q., Chan D., Cheah K.S.E. (2018). Inhibiting the integrated stress response pathway prevents aberrant chondrocyte differentiation thereby alleviating chondrodysplasia. Elife.

[bb1180] Wang H., Zheng C., Lu W., He T., Fan J., Wang C., Jie Q., Chan D., Cheah K.S.E., Yang L. (2022). Hedgehog signaling orchestrates cartilage-to-bone transition independently of smoothened. Matrix Biol..

[bb1185] Wang K., Ma C., Feng J.Q., Jing Y. (2022). The emerging role of cell transdifferentiation in skeletal development and diseases. Int. J. Mol. Sci..

[bb1190] Warman M.L., Abbott M., Apte S.S., Hefferon T., McIntosh I., Cohn D.H., Hecht J.T., Olsen B.R., Francomano C.A. (1993). A type X collagen mutation causes Schmid metaphyseal chondrodysplasia. Nat. Genet..

[bb1195] Wilson R., Norris E.L., Brachvogel B., Angelucci C., Zivkovic S., Gordon L., Bernardo B.C., Stermann J., Sekiguchi K., Gorman J.J., Bateman J.F. (2012). Changes in the chondrocyte and extracellular matrix proteome during post-natal mouse cartilage development. Mol. Cell. Proteomics.

[bb1200] Xiong J., Onal M., Jilka R.L., Weinstein R.S., Manolagas S.C., O’Brien C.A. (2011). Matrix-embedded cells control osteoclast formation. Nat. Med..

[bb1205] Yagami K., Suh J.Y., Enomoto-Iwamoto M., Koyama E., Abrams W.R., Shapiro I.M., Pacifici M., Iwamoto M. (1999). Matrix GLA protein is a developmental regulator of chondrocyte mineralization and, when constitutively expressed, blocks endochondral and intramembranous ossification in the limb. J. Cell Biol..

[bb1210] Yakar S., Werner H., Rosen C.J. (2018). Insulin-like growth factors: actions on the skeleton. J. Mol. Endocrinol..

[bb1215] Yamasaki A., Itabashi M., Sakai Y., Ito H., Ishiwari Y., Nagatsuka H., Nagai N. (2001). Expression of type I, type II, and type X collagen genes during altered endochondral ossification in the femoral epiphysis of osteosclerotic (oc/oc) mice. Calcif. Tissue Int..

[bb1220] Yan B., Zhang Z., Jin D., Cai C., Jia C., Liu W., Wang T., Li S., Zhang H., Huang B., Lai P., Wang H., Liu A., Zeng C., Cai D., Jiang Y., Bai X. (2016). mTORC1 regulates PTHrP to coordinate chondrocyte growth, proliferation and differentiation. Nat. Commun..

[bb1225] Yang Y., Topol L., Lee H., Wu J. (2003). Wnt5a and Wnt5b exhibit distinct activities in coordinating chondrocyte proliferation and differentiation. Development.

[bb1230] Yang G., Zhu L., Hou N., Lan Y., Wu X.-M., Zhou B., Teng Y., Yang X. (2014). Osteogenic fate of hypertrophic chondrocytes. Cell Res..

[bb1235] Yang L., Tsang K.Y., Tang H.C., Chan D., Cheah K.S. (2014). Hypertrophic chondrocytes can become osteoblasts and osteocytes in endochondral bone formation. Proc. Natl. Acad. Sci. U. S. A..

[bb1240] Yeung P., Cheng K.H., Yan C.H., Chan B.P. (2019). Collagen microsphere based 3D culture system for human osteoarthritis chondrocytes (hOACs). Sci. Rep..

[bb1245] Yoshida C.A., Yamamoto H., Fujita T., Furuichi T., Ito K., Inoue K., Yamana K., Zanma A., Takada K., Ito Y., Komori T. (2004). Runx2 and Runx3 are essential for chondrocyte maturation, and Runx2 regulates limb growth through induction of Indian hedgehog. Genes Dev..

[bb1250] Yoshioka C., Yagi T. (1988). Electron microscopic observations on the fate of hypertrophic chondrocytes in condylar cartilage of rat mandible. J. Craniofac. Genet. Dev. Biol..

[bb1255] Yu L., Liu H., Yan M., Yang J., Long F., Muneoka K., Chen Y. (2007). Shox2 is required for chondrocyte proliferation and maturation in proximal limb skeleton. Dev. Biol..

[bb1260] Zanotti S., Canalis E. (2013). Interleukin 6 mediates selected effects of notch in chondrocytes. Osteoarthr. Cartil..

[bb1265] Zelzer E., Glotzer D.J., Hartmann C., Thomas D., Fukai N., Soker S., Olsen B.R. (2001). Tissue specific regulation of VEGF expression during bone development requires Cbfa1/Runx2. Mech. Dev..

[bb1270] Zelzer E., Mamluk R., Ferrara N., Johnson R.S., Schipani E., Olsen B.R. (2004). VEGFA is necessary for chondrocyte survival during bone development. Development.

[bb1275] Zhang M., Xie R., Hou W., Wang B., Shen R., Wang X., Wang Q., Zhu T., Jonason J.H., Chen D. (2009). PTHrP prevents chondrocyte premature hypertrophy by inducing cyclin-D1-dependent Runx2 and Runx3 phosphorylation, ubiquitylation and proteasomal degradation. J. Cell Sci..

[bb1280] Zheng Q., Zhou G., Morello R., Chen Y., Garcia-Rojas X., Lee B. (2003). Type X collagen gene regulation by Runx2 contributes directly to its hypertrophic chondrocyte-specific expression in vivo. J. Cell Biol..

[bb1285] Zhou Z., Apte S.S., Soininen R., Cao R., Baaklini G.Y., Rauser R.W., Wang J., Cao Y., Tryggvason K. (2000). Impaired endochondral ossification and angiogenesis in mice deficient in membrane-type matrix metalloproteinase I. Proc. Natl. Acad. Sci..

[bb1290] Zhou B.O., Yue R., Murphy M.M., Peyer J.G., Morrison S.J. (2014). Leptin-receptor-expressing mesenchymal stromal cells represent the main source of bone formed by adult bone marrow. Cell Stem Cell.

[bb1295] Zhou X., von der Mark K., Henry S., Norton W., Adams H., de Crombrugghe B. (2014). Chondrocytes transdifferentiate into osteoblasts in endochondral bone during development, postnatal growth and fracture healing in mice. PLoS Genet..

[bb1300] Zuo C., Wang L., Kamalesh R.M., Bowen M.E., Moore D.C., Dooner M.S., Reginato A.M., Wu Q., Schorl C., Song Y., Warman M.L., Neel B.G., Ehrlich M.G., Yang W. (2018). SHP2 regulates skeletal cell fate by modifying SOX9 expression and transcriptional activity. Bone Res..

